# Chemically induced revitalization of damaged hepatocytes for regenerative liver repair

**DOI:** 10.1016/j.isci.2023.108532

**Published:** 2023-11-23

**Authors:** Pengyan Lin, Yunfei Bai, Xinxin Nian, Jun Chi, Tianzhe Chen, Jing Zhang, Wenpeng Zhang, Bin Zhou, Yang Liu, Yang Zhao

**Affiliations:** 1 State Key Laboratory of Natural and Biomimetic Drugs, Ministry of Education Key Laboratory of Cell Proliferation and Differentiation, Beijing Key Laboratory of Cardiometabolic Molecular Medicine, Institute of Molecular Medicine, College of Future Technology, Peking University, Beijing 100871, China; 2 Plastech Pharmaceutical Technology Co., Ltd, Nanjing 210043, China; 3 Peking-Tsinghua Center for Life Science, Peking University, Beijing 100871, China; 4 State Key Laboratory of Toxicology and Medical Countermeasures, Beijing Institute of Pharmacology and Toxicology, Beijing 100850, China; 5 New Cornerstone Science Laboratory, State Key Laboratory of Cell Biology, Shanghai Institute of Biochemistry and Cell Biology, Center for Excellence in Molecular Cell Science, Chinese Academy of Sciences, University of Chinese Academy of Sciences, Shanghai, China

**Keywords:** Pharmacology, Chemical compounds, Natural sciences, Chronic liver injury, Biological sciences, Cell revitalization, Physiology, Liver regeneration

## Abstract

In prolonged liver injury, hepatocytes undergo partial identity loss with decreased regenerative capacity, resulting in liver failure. Here, we identified a five compound (5C) combination that could restore hepatocyte identity and reverse the damage-associated phenotype (e.g., dysfunction, senescence, epithelial to mesenchymal transition, growth arrest, and pro-inflammatory gene expression) in damaged hepatocytes (dHeps) from CCl4-induced mice with chronic liver injury, resembling a direct chemical reprogramming approach. Systemic administration of 5C in mice with chronic liver injury promoted hepatocyte regeneration, improved liver function, and ameliorated liver fibrosis. The hepatocyte-associated transcriptional networks were reestablished with chemical treatment as revealed by motif analysis of ATAC-seq, and a hepatocyte-enriched transcription factor, Foxa2, was found to be essential for hepatocyte revitalization. Overall, our findings indicate that the phenotype and transcriptional program of dHeps can be reprogrammed to generate functional and regenerative hepatocytes by using only small molecules, as an alternative approach to liver repair and regeneration.

## Highlights

• 5 compounds (5C) reverses hepatocyte injury (e.g., identity loss, senescence, EMT)
• 5C restores function, boosts proliferation in hepatocytes *in vitro* and *in vivo*
• 5C alleviates fibrosis, promotes liver repair and regeneration post-chronic injury
• Foxa2 mediates chemically induced restoration of hepatocyte transcriptional program

## Graphical abstract

**Figure:**
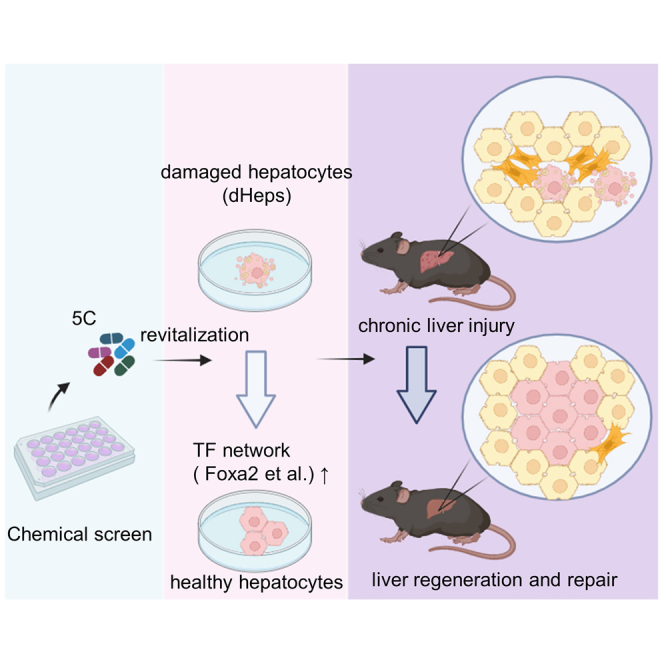

## Introduction

The liver harbors remarkable regenerative capacity and performs multiple physiological functions.^1^^,^^2^^,^^3^^,^^4^ Following partial hepatectomy or toxin-induced injury, the liver can efficiently regenerate through the rapid proliferation of hepatocytes.^4^ Despite the liver’s remarkable capacity for regeneration, this ability becomes overwhelmed and dysfunctional following severe chronic or acute liver injury.^5^ Therefore, it is beneficial to develop methods to identify cell sources and underlying mechanisms of regeneration.

Previous studies of the progression of liver diseases have reported that hepatocytes can undergo cell state/phenotype changes: for instance, a fibrosis-activated hepatocyte transcriptomic program promotes hepatocyte dysfunction and directs the progression of non-alcoholic steatohepatitis (NASH).^6^ Loss of hepatic identity has been reported for cells that undergo epithelial-mesenchymal transition (EMT).^7^ Similarly, core hepatic transcription factors (TFs) and hepatocyte-specific genes were shown to be repressed in patients with cirrhosis of various etiologies.^8^ In addition, an earlier study examining chronic liver injury showed that a network of hepatocyte-enriched TFs is downregulated in hepatocytes, which led to the shutdown of hepatocyte functions that is not naturally reversible, even following the elimination of the liver injury.^9^

Cell fates/states can be converted into other lineages through transdifferentiation as an approach to generate functional cells for regenerative medicine applications.^10^ Several previous reports have described the direct induction of functional hepatocytes from fibroblasts by the exogenous expression of a combination of hepatocyte-enriched TFs (e.g., Foxa2, Hnf1a, and Hnf4a) or chemical induction of the endogenous hepatic TFs via an induced plastic state.^11^^,^^12^^,^^13^^,^^14^^,^^15^ Thus, it could be attractive to know whether the damage-associated state/phenotype in dHeps can be reversed by using a chemical reprogramming-like approach to establish a functional hepatocyte identity for liver repair and regeneration.

In this study, we identified 5C that can restore hepatocyte identity and functions while reversing the injured hepatocyte state/phenotype, including mesenchymal transition, senescence, and inflammation. dHeps were reprogrammed in epigenetic levels and regained a hepatocyte-specific transcriptional circuit, involving the activation of Foxa2, a hepatocyte-enriched transcription factor. The chemical compounds described here were effective in chronic mouse models of liver injury *in vivo*, suggesting the therapeutic potential of this approach for regeneration and functional recovery of organs.

## Results

### Identification of chemical compounds to revitalize damaged hepatocytes with identity loss

To test whether the reversion of damage-associated phenotypes and restoration of cell identity and function (revitalization) in dHeps can be induced with chemical compounds, we first characterized the phenotypes of dHeps from Carbon tetrachloride (CCl4)-induced chronic liver injury model mice (C57BL/6 background). CD45^−^ liver cells were isolated from CCl4-injured mice for 12 weeks and healthy mice, respectively, and purified by magnetic-activated cell sorting (MACS). Single cell RNA sequencing (scRNA-seq) was performed following a 10X Chromium workflow. Hepatocyte populations of liver cells were recognized with highly specific hepatic markers including *Arg1*, *Tat*, *Alb*, and so forth and their heterogeneity was analyzed by using unsupervised clustering, which revealed significant upregulation of mesenchymal, cell death, and inflammatory chemokine genes (*Igfbp3*, *Cx3cl1*, *Ecm1*, *Lgals3*, *Col1a1*, *Col1a2*, *Acta2*, *Des*, *Pdgfra*, and *Pdgfrb*) concurrent with the downregulation of hepatocyte-enriched transcription factors and apoptosis-inhibiting factors (*Hnf1a*, *Hnf1b*, *Hnf4a*, *Foxa3*, *Foxa2*, *Foxa1*, *Xiap*, *Birc2*, *Birc3*) in CCl4-injured hepatocytes, as compared to uninjured control hepatocytes (Figure S1). We next applied an AAV-TBG-Cre: Rosa-LSL-tdTomato hepatic cell tracing system to track the lineage of hepatocytes during CCl4-induced liver injury. Rosa-LSL-tdTomato mice were intravenously injected with adeno-associated virus (AAV) serotype 8 particles expressing Cre recombinase driven by the liver-specific thyroxine-binding globulin (TBG) promoter, which efficiently expressed tdTomato in hepatocytes. z stack confocal images of injured liver sections uncovered tdTomato^+^ cells expressed Desmin (a hepatic stellate cell marker) and Collagen (a fibroblast marker) with mesenchyme-like morphology (Figure S2). These findings suggested that hepatocytes underwent partial loss of hepatocyte cell identity and gained damage-associated phenotypes including EMT, apoptosis, and inflammation during CCl4-induced liver injury.

**Figure 1:**
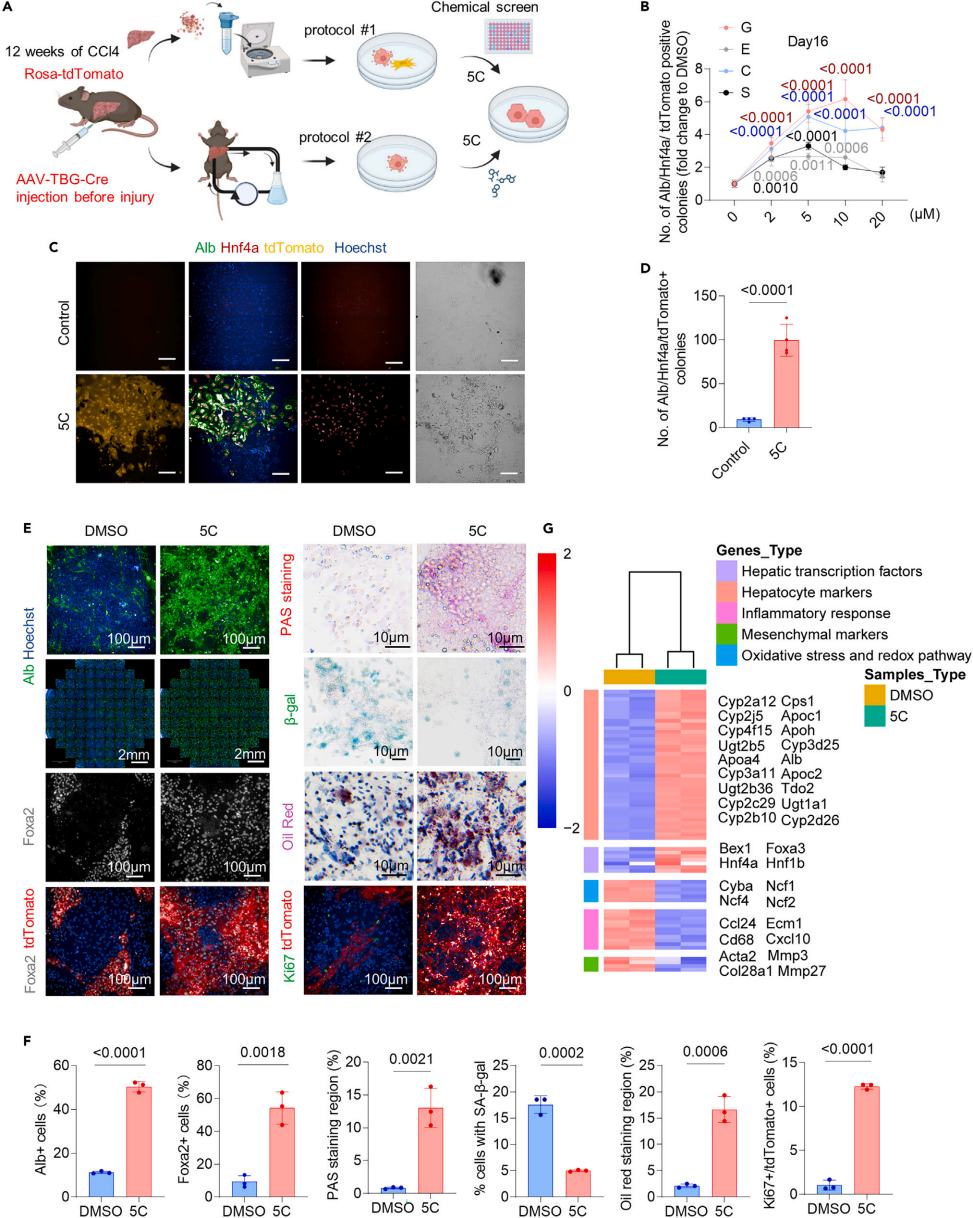
Identification of chemical compounds to revitalize dHeps with identity loss (A) Schematic of small molecule screen to revitalize hepatocyte-like cells from dHeps isolated from CCl4-injured mice. (B) Numbers of albumin and Hnf4a double-positive epithelial cell colonies labeled by AAV-TBG-Cre: Rosa-LSL-tdTomato hepatic cell tracing system after transduction with small molecules in the presence of FSK on day 16. Results are means ± SD for three biological replicates. Two-way ANOVA was performed to determine statistical significance. Salidroside (S), CID755673 (C), GSK429286A (G), and ETC-1002 (E). (C and D) Representative images of morphology (C) and numbers (D) of albumin-positive and Hnf4a-positive epithelial cell colonies labeled by AAV-TBG-Cre: Rosa-LSL-tdTomato hepatic cell tracing system after transduction with 5C on day 16. Scale bar, 100 μm. (E) The morphology of Alb-positive (green), Ki67-positive (green), and Foxa2-positive (gray) hepatocytes after the treatment of 5C, and DMSO in dHeps isolated from a CCl4-induced mouse model. Nuclei are stained with DAPI (blue). PAS staining was performed to measure glycogen storage, Oil red staining was performed to measure lipid synthesis, and SA-β-gal staining showed that senescence decreased after the treatment of chemical compounds. (F) The percentage of Alb-positive hepatocytes, Foxa2-positive hepatocytes, Ki67 and tdTomato double-positive hepatocytes, Periodic-Acid-Schiff (PAS)-positive cell regions, Oil red-positive cell regions, SA-β-gal-positive cells after the treatment of 5C, and DMSO. Results are means ± SD for 3 biological replicates. Significance was assessed using an unpaired Student’s *t* test. (G) Heatmaps showing the upregulation of genes associated with hepatic TFs, hepatocyte markers, and the downregulation of genes associated with inflammatory response, mesenchymal markers, and oxidative stress and redox pathway after the treatment of 5C compared with DMSO in dHeps isolated from CCl4-induced mouse model. Log2 fold-change>1. The color bar indicates gene expression in Log2 scale.

Then, dHeps were isolated from CCl4-injured mice for chemical screening (Figure 1A), and the AAV-TBG-Cre: Rosa-LSL-tdTomato cell tracing system was also applied to discriminate injured hepatocytes from other liver mesenchymal cells. Chemical screens were carried out in the presence of forskolin (FSK), an adenylate cyclase (AC), since FSK was known to be beneficial to hepatocyte maintenance,^16^ which was also validated with qPCR assay (Figure S3A). A subsequent screen of >6,000 compounds from the kinase library, epigenetic library, and approved drug repurposing library for their effects on inducing Albumin (Alb)^+^, Hnf4a^+^, and tdTomato^+^ hepatocyte-like colonies from dHeps, identified that 4 compounds could each promote the formation of hepatocyte-like colonies: the protein kinase D inhibitor CID755673; the ROCK inhibitor GSK429286A; the AMPK activator ETC-1002; and a phenylpropanoid glycoside from *Rhodiola Rosea* L. (Salidroside) (Figure 1B). We further found that the combination of these 5 compounds (5C), i.e., 4 hit compounds plus FSK, can significantly promote the formation of tdTomato^+^ hepatocytes-like colonies compared with that in the DMSO controls (∼10-fold), as were single compound plus FSK (Figures 1B–1D).

In addition, another dHeps isolation protocol that enriches hepatocyte-like cells was also applied to examine the alteration of cell phenotypes with 5C compounds. Up to 95% of cells isolated from CCl4-injured AAV-TBG-Cre: Rosa-LSL-tdTomato mice were tdTomato+, indicating the enrichment of hepatocyte-derived cells, e.g., dHeps (Figure S3B). We have also assessed the differences between primary hepatocytes isolated from healthy mice and those isolated from CCl4-injured mice. Morphological observations showed that EMT and cell death were more prominent in primary hepatocytes isolated from CCl4-injured mice compared to those from healthy mice *in vitro* (Figure S3C). Additionally, fewer Alb^+^ and Foxa2^+^ cells were detected in primary hepatocytes isolated from CCl4-injured mice after 16 days *in vitro* compared with that in hepatocytes from healthy mice cultured under the same conditions (Figures S3D and S3E). Analysis by qPCR indicated that hepatocytes isolated from injured liver displayed significant upregulation of mesenchymal identity-, inflammatory-, apoptosis-, and senescence-related genes (*Timp1*, *Acta2*, *Col1a1*, *Cd93*, *Serpine1*, *Mmp3*, and *P16*) after 16 days *in vitro*, while hepatic TF genes (*Alb, Hnf4a*, *Hnf6*, *Foxa2*) were downregulated compared with hepatocytes isolated from healthy mice under the same conditions (Figure S3F).

We further found that 5C resulted in sustained Alb expression in dHeps and increased the proportions of Alb^+^ cells with hepatocyte-like morphology (∼5 times greater than that in controls) after 16 days. In line with the enhanced Alb expression, immunostaining indicated that 5C increased the proportions of Ki67^+^ (∼12-fold) and Foxa2^+^ (∼5-fold) tdTomato^+^ hepatocyte-like cells compared with the DMSO control and improved the recovery of the glycogen storage and lipid synthesis function compared with DMSO control, indicating the restore of hepatocyte identity, regenerative potential, and function with chemical treatment. Additionally, 5C reduced senescence-associated β-galactosidase activity, indicating the alleviation of senescence of dHeps (Figures 1E and 1F).

Gene expression profiles of dHeps after treatment with 5C, or DMSO control after 16 days were analyzed by RNA sequencing (RNA-seq). RNA-seq analysis of dHeps showed that the expression of hepatocyte-enriched TFs such as *Hnf1b*, *Hnf4a*, and *Foxa3*, as well as drug metabolism enzymes such as *Cyp3a11*, *Ugt1a1*, and *Cyp2c29,* was upregulated in the 5C groups, while mesenchymal markers and inflammatory-related genes including *Acta2*, *Mmp3*, *Cxcl10*, *Ecm1*, *Ccl24*, and *Cd68* were down-regulated, as were oxidative stress-related genes *Ncf2*, *Ncf1* compared with their expression in DMSO control (Figures 1G and S4A). GO analysis and KEGG analysis indicated genes downregulated in the 5C group compared to the DMSO control cells were enriched for terms associated with mesenchymal transition, inflammation, oxidative stress, and so forth, while upregulated genes were enriched for the terms associated with hepatocyte identity and function (Figures S4B and S4C). Overall, these indicate a reversion of comprehensive phenotypes of dHeps with 5C treatment. Thus, we termed such a process of phenotypic reversal in dHeps as hepatocyte revitalization.

### 5 compounds induced the revitalization of hepatocytes injured by transforming growth factor β treatment

**Figure 2:**
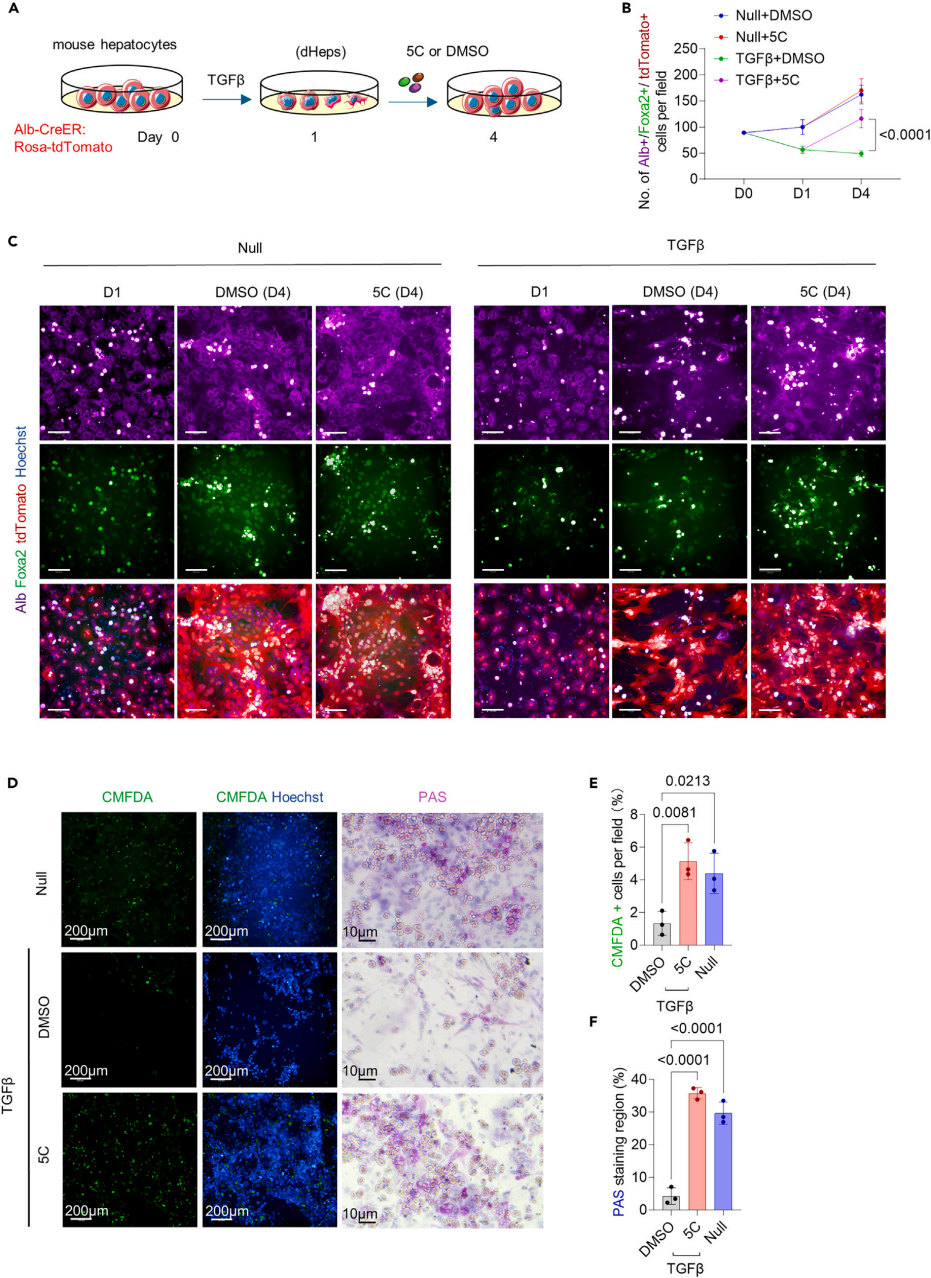
5C induced the revitalization of hepatocytes injured by transforming growth factor β (TGF-β) treatment (A) Primary hepatocytes isolated from mice expressing the Alb-CreER: Rosa-LSL-tdTomato hepatic cell tracing system were treated for 24h with 5 ng/mL TGF-β, followed by 5C or DMSO (control). (B) Growth curves of Foxa2^+^/Alb^+^/tdTomato^+^ hepatocytes cultured with 5C or DMSO after treatment with or without TGF-β treatment. Results are means ± SD for three biological replicates. Two-way ANOVA was performed to determine statistical significance. Positive controls healthy cells without TGF-β treatment (Null). (C) Representative images of morphology of Foxa2^+^/Alb^+^/tdTomato^+^ hepatocytes cultured with 5C or DMSO on day 4 after treatment with or without TGF-β treatment. Scale bar, 100 μm. Positive controls healthy cells without TGF-β treatment (Null). (D and F) Representative images (D) with quantitative analysis of the percentage of CMFDA-positive (green) (E), and PAS-positive hepatocytes (F) after treatment with 5C, or DMSO at day 6 in dHeps induced by 24h exposure to TGF-β. Nuclei are stained with Hoechst (blue). PAS staining was performed to measure glycogen storage. Results are means ± SD for 3 biological replicates. Significance was assessed using one-way ANOVA. Positive controls healthy cells without TGFβ treatment (Null).

TGF-β, a prominent stimulus of hepatocyte injury in most liver diseases leads to EMT and apoptosis in hepatocytes,^17^^,^^18^^,^^19^ similar to the hepatocyte phenotypes defined by scRNA-seq in CCl4-induced chronic liver injury (Figure S1). Therefore, we also established an *in vitro* model of hepatocyte injury by the treatment with TGF-β, to further illustrate the effects of 5C on dHeps. For this model, tdTomato-labeled mouse primary hepatocytes were isolated from Alb-CreER: Rosa-tdTomato mice, which constitutively expressed tdTomato, initiated by tamoxifen-inducible Cre recombinase expression driven by the Alb promoter, resulting in the efficient expression of tdTomato in hepatocytes after two weeks of tamoxifen treatment (Figure 2A). Hepatocyte culture were treated with TGF-β for 24 h, and subsequently with 5C/DMSO for another 3 days. We observed a dramatic increase in the number of Foxa2^+^/Alb^+^/tdTomato^+^ cells among dHeps treated with 5C compared with that in DMSO control cultures, which showed a significant decrease in the number of Foxa2^+^/Alb^+^/tdTomato^+^ cells and could not self-recover after withdrawal of TGF-β. These results indicated that 5C could promote the expansion and survival of dHeps induced with TGF-β treatment (Figures 2B and 2C).

To then validate that 5C stimulated the revitalization of TGF-β-induced dHeps instead of including the expansion of a healthy hepatocyte contaminant population, we compared the effects of 5C in normal hepatocytes without TGF-β treatment to that in TGF-β-induced dHeps over 4 days growth curve. We found that the numbers of Foxa2^+^/Alb^+^/tdTomato^+^ hepatocytes were comparable between normal hepatocytes without TGF-β treatment treated for 3 days with 5C and DMSO controls for normal hepatocytes without TGF-β treatment. In contrast, the numbers of Foxa2^+^/Alb^+^/tdTomato^+^ hepatocytes significantly increased (∼2.37-fold) in 5C-treated dHeps, compared with that in DMSO control cultures (Figures 2B and 2C). These results indicated that 5C treatment could significantly reverse the arrested growth and loss of cell identity (indicated by the Alb and Foxa2 downregulation) resulting from TGF-β exposure rather than promoting the expansion of the healthy hepatocytes.

In line with the increased Foxa2^+^/Alb^+^/tdTomato^+^ hepatocytes numbers by 5C treatment, immunostaining indicated that the numbers of Hnf4a^+^/Alb^+^/tdTomato^+^ hepatocytes significantly increased in 5C-treated dHeps induced by TGF-β, compared with DMSO control cultures (Figure S5). In addition, intracellular 5-chloromethylfluorescein diacetate (CMFDA) staining^20^^,^^21^ and PAS staining indicated that 5C-treated dHeps restored hepatic polarity and glycogen storage, which were impaired after injury (Figures 2E and 2F). These results further supported that 5C could stimulate hepatocyte revitalization *in vitro* after TGF-β-induced liver injury, including the restoration of hepatic gene expression, proliferative capacity, polarization, and glycogen storage function.

### 5 compounds promotes hepatocyte expansion and survival *in vivo* after CCl4-induced liver injury

**Figure 3:**
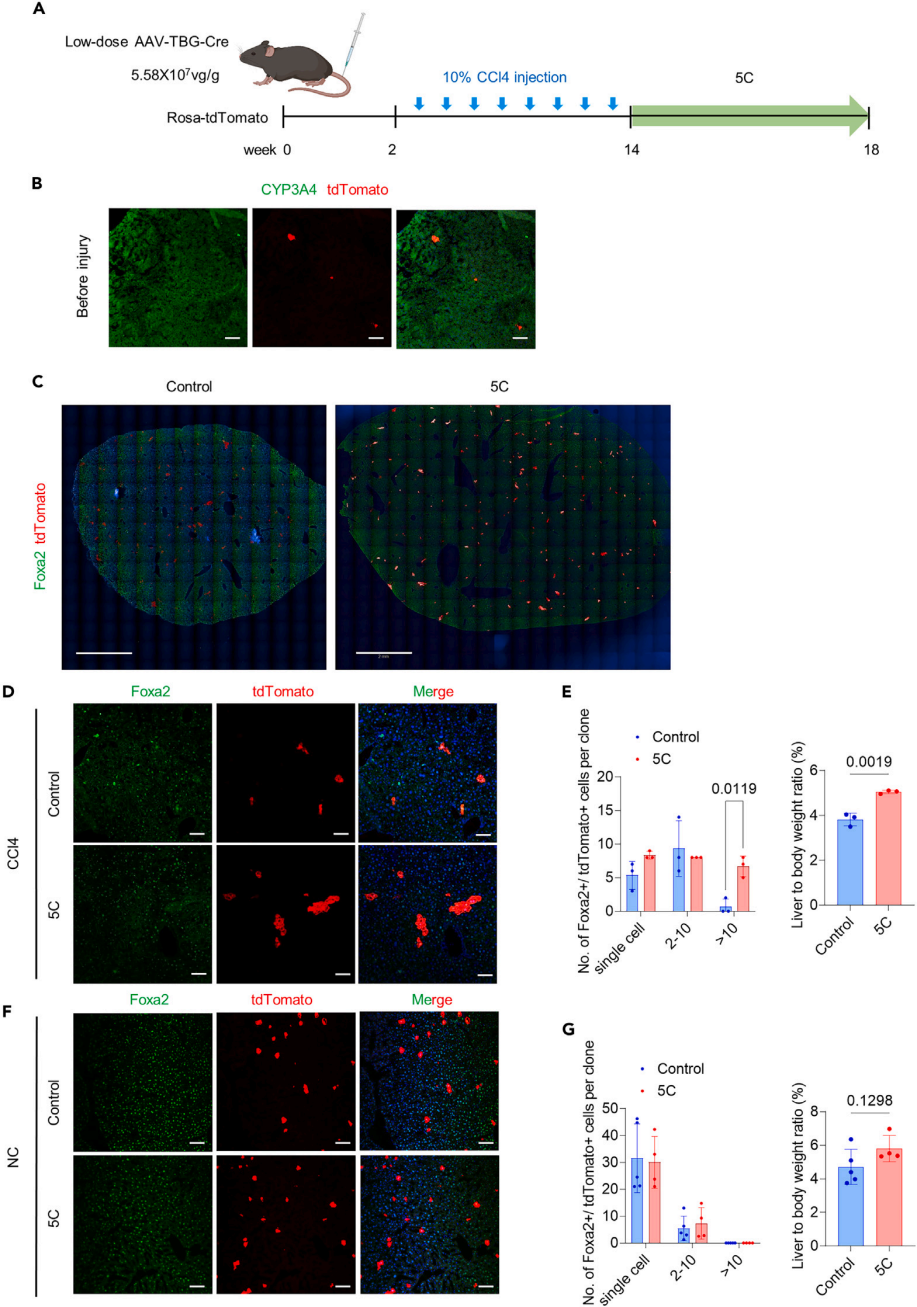
5C promote hepatocytes expansion and survival after CCl4-induced liver injury (A) Schematic of low-density lineage tracing by applied AAV-TBG-Cre: Rosa-LSL-tdTomato hepatic cell tracing system and used low vector doses to assess hepatocyte expansion and survival with chemical compounds or solvent control after CCl4-induced liver injury. (B) Immunofluorescence staining with antibodies for hepatocyte markers CYP3A4 on cryosections from AAV-TBG-Cre: Rosa26-LSL-tdTomato lineage-tracing mice before CCl4 treatment. Nuclei are stained with DAPI (blue). Scale bar, 100 μm. (C) Immunofluorescence staining with antibodies for hepatocyte markers Foxa2 on cryosections from AAV-TBG-Cre: Rosa26-LSL-tdTomato lineage-tracing mice with 5C, and solvent control after CCl4 treatment. Nuclei are stained with DAPI (blue). Scale bar, 2mm. (D and E) The morphology and numbers of tdTomato-expressing epithelial cell colonies staining positive for Foxa2 per slide in the right liver lobe from three mice per group two-way ANOVA was performed to determine statistical significance. Nuclei are stained with DAPI (blue). Scale bar, 100 μm. Quantification of liver/body mass ratios in 5C-treated mice in chronic liver fibrosis models (n = 3) compared to controls (n = 3). Significance was assessed using an unpaired Student’s *t* test. (F and G) The morphology and numbers of tdTomato-expressing epithelial cell colonies staining positive for Foxa2 per slide in the right liver lobe from 5C-treated mice (n = 4) and controls (n = 5). Two-way ANOVA was performed to determine statistical significance. Nuclei are stained with DAPI (blue). Scale bar, 100 μm. Quantification of liver/body mass ratios in 5C-treated mice without injury (n = 4) compared to controls (n = 5). Significance was assessed using an unpaired Student’s *t* test.

We next tested whether the induction of cell revitalization could promote hepatic regeneration *in vivo*. To investigate the clonal expansion of hepatocytes in CCl4-induced liver injury, we applied an AAV-TBG-Cre: Rosa-LSL-tdTomato hepatic cell tracing system with low vector doses to induce low-density lineage tracing^22^ (Figure 3A). Under these conditions, tdTomato was expressed in less than 0.24% of hepatocytes after two weeks of AAV injection (Figure 3B), so that hepatocyte expansion could be detected by measuring the size of tdTomato+ hepatocyte clusters. These mice were then injured with CCl4 twice per week for 12 weeks, then treated with 5C or the solvent control for four weeks after AAV vector injection. Immunostaining of liver tissue showed that the number of cloned hepatocyte clusters (>10 cells) significantly increased by ∼10-fold in 5C-treated mice compared with that in the solvent controls, along with a ∼1.31-fold increase in liver/body mass ratio (Figures 3C–3E), suggesting that 5C could enhance the regenerative capacity of hepatocytes *in vivo* after CCl4-induced liver injury.

In addition, we also tested the effect of 5C treatment in healthy mice with low-density hepatic lineage tracing to assess whether 5C promoted the expansion of healthy hepatocytes *in vivo*. Rosa-tdTomato mice were intravenously injected with low vector doses of AAV, followed by a washout period of 2 weeks. These mice were then treated with 5C or the solvent control for four weeks after AAV vector injection without injury. Immunostaining of liver tissue showed that the numbers of cloned hepatocyte clusters was not significantly different between 5C and the solvent control (Figures 3F and 3G). These results indicated that 5C could not promote the expansion of healthy hepatocytes without CCL4-induced injury.

### Restoration of regenerative potential in damaged hepatocytes traced with Col1a2 in mice with chronic liver injury

**Figure 4:**
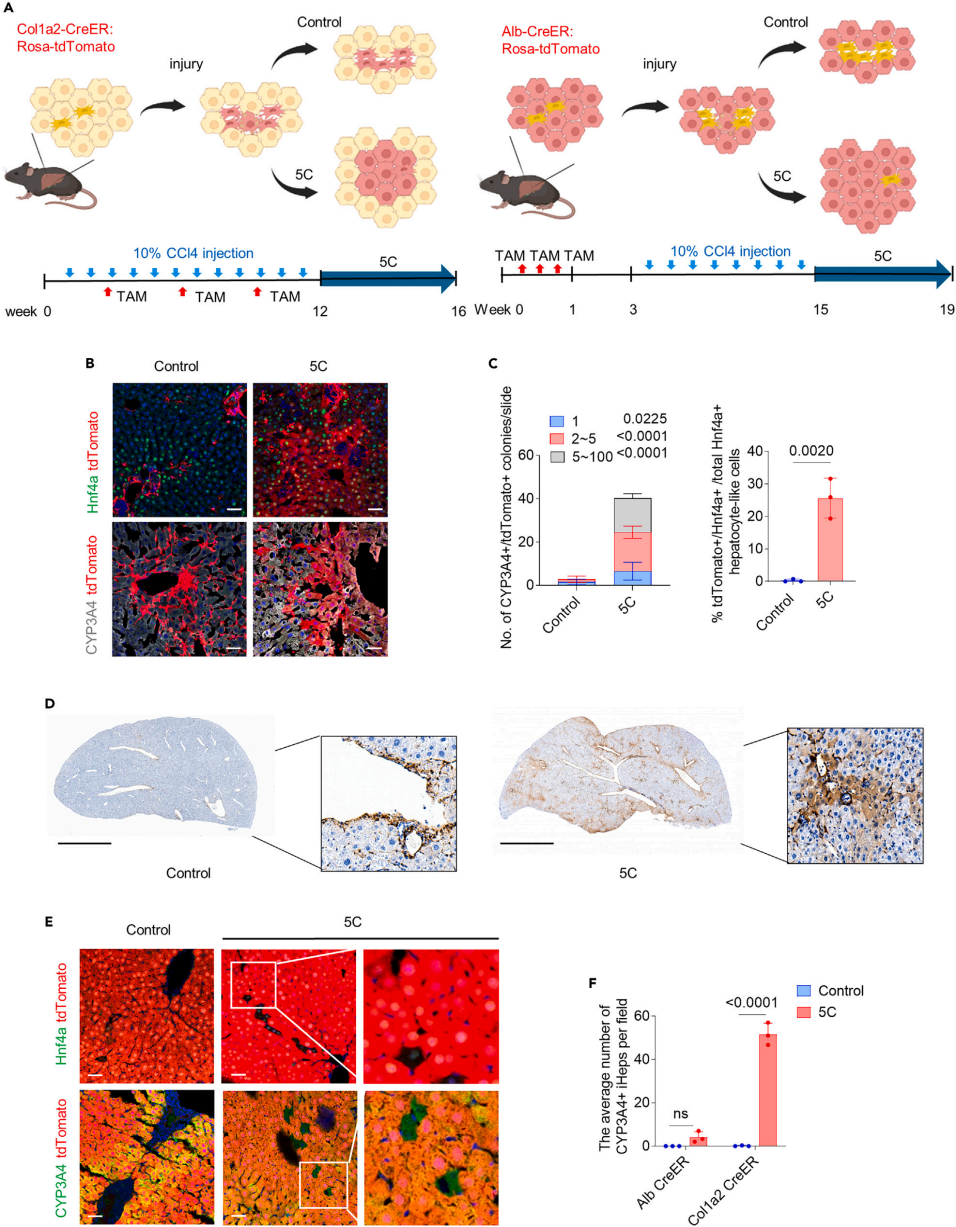
Restoration of regenerative potential in dHeps traced with Col1a2 in mice with chronic liver injury (A) Schematic of the chronic liver fibrosis model (10% CCl4 injection twice weekly for 12 weeks) to assess the *in vivo* hepatocyte expansion of dHeps with 5C in Col1a2-CreER: Rosa26-LSL-tdTomato and Alb-CreER: Rosa26-LSL-tdTomato lineage-tracing mice, which was created in BioRender.com. (B) Immunofluorescence staining with antibodies for hepatocyte markers CYP3A4 and Hnf4a on cryosections from Col1a2-CreER: Rosa26-LSL-tdTomato lineage-tracing mice with 5C after CCl4 treatment. Nuclei are stained with DAPI (blue). Scale bar, 50 μm. (C) Numbers of tdTomato-expressing epithelial cell colonies staining positive for CYP3A4 per slide in the right liver lobe from four mice per group. Two-way ANOVA was performed to determine statistical significance. Percentage of average tdTomato-expressing epithelial-like cells staining positive for Hnf4a in the right liver lobe obtained from three mice per group. Significance was assessed using an unpaired Student’s *t* test. (D) Immunohistochemistry with antibodies for RFP on paraffin-embedded sections Col1a2-CreER: Rosa26-LSL-tdTomato lineage-tracing mice with 5C after liver injuries. The section was detected using a ready-to-use Goat Anti-Rat lgG HRP-liked antibody. Scale bar, 3 mm. (E) Immunofluorescence staining with antibodies (green) for the hepatocyte markers CYP3A4 and Hnf4a on cryosections from Alb-CreER: Rosa26-LSL-tdTomato lineage-tracing mice with 5C after CCl4 treatment. Nuclei are stained with DAPI (blue). Scale bar, 50 μm. (F) The average number of CYP3A4 positive induced hepatocyte-like cells per field from Alb-CreER: Rosa26-LSL-tdTomato lineage-tracing mice or Col1a2-CreER: Rosa26-LSL-tdTomato lineage-tracing mice with 5C and solvent control after CCl4 treatment. Two-way ANOVA was performed to determine statistical significance.

We next examined whether 5C could promote hepatocyte regeneration from dHeps after chronic liver injury *in vivo*. Col1a2-CreER: Rosa26-tdTomato mice were then used to constitutively label cells expressing Col1a2 after injury, including Col1a2-traced dHeps, to examine their behaviors after 5C administration *in vivo*. Although this lineage tracing system could also label liver myofibroblasts after injury,^23^^,^^24^ we further applied Alb-CreER: Rosa-tdTomato mice to label all hepatocyte-derived cells, as counterparts to assess the possibility of hepatic reprogramming from myofibroblasts (Figure 4A).

After CCl4 injection for 12 weeks, Col1a2-CreER: Rosa26-tdTomato mice were systemically injected with 5C or solvent control for an additional four weeks (Figure 4A). To label liver Col1a2-traced cells, tamoxifen was fed 3 times during chronic liver fibrosis. Immunostaining of liver biopsies from 5C-treated mice showed the emergence of hepatocyte-like colonies co-expressing tdTomato with hepatocyte markers such as Hnf4a and CYP3A4, with∼16-fold higher colony numbers than that in the solvent control (Figures 4B and 4C). Moreover, those induced hepatocytes constituted as much as 10%–20% of the total hepatocyte population in the analyzed lobe (Figures 4C and 4D). Notably, those induced hepatocytes tended to form large clusters (*i.e*., up to 100 cells) in 5C-treated mice, indicating their *in vivo* expansion (Figure 4C). To evaluate the possible “leakiness” of the Col1a2 promoter activity prior to liver injury and 5C treatment, Col1a2-CreER: Rosa-mice without liver injury were fed with 3 doses of tamoxifen, followed by a two-week washout period, then subsequently treated with 5C or the solvent control for four weeks (Figure S6A). Immunostaining of liver sections indicated that all the Col1a2-CreER-labeled cells were Desmin^+^ mesenchymal cells, while no Hnf4a^+^ or CYP3A4^+^ and tdTomato^+^ double-positive hepatocyte-like cells were detected in either 5C treatment or control groups (Figure S6B). This finding supports that the Col1a2-CreER system labeled mesenchymal cells, and not hepatocytes without injury, and that the chemical treatment did not induce the “leaky” Col1a2 promoter activity. These results suggested that 5C could promote the formation of hepatocyte-like cell colonies from Col1a2-traced cells after CCl4-induced liver injury.

To figure out whether the induction of hepatocyte-like colonies is restricted to only CCl4-induced liver injury models, we also tested whether 5C could induce hepatocyte revitalization in a cholestasis-associated liver injury model induced by the dietary administration of 3, 5-methoxycarbonyl-1,4-dihydrocollidine (DDC). In line with our findings in CCl4-induced liver injury, immunostaining of liver biopsies from 5C-treated mice with DDC-induced liver injury showed the formation of tdTomato^+^ and CYP3A4^+^ hepatocyte-like colonies, with colony numbers ∼10-fold higher than that in the solvent control (Figure S7). These results indicated that 5C could also promote the regeneration of hepatocyte-like colonies from Col1a2-traced cells during DDC-induced liver injury.

To assess whether revitalized hepatocytes-like cells were in fact transdifferentiated from Col1a2-traced liver myofibroblasts or Col1a2-traced hepatocyte-derived cells, Alb-CreER: Rosa-tdTomato mice were used to delineate the specific contributions of hepatocytes and non-parenchymal cells (NPCs) to the formation of hepatocyte-like cells after 5C treatment. Tamoxifen was administered to Alb-CreER: Rosa-tdTomato mice before injury to induce tdTomato labeling in hepatocytes while NPCs remained tdTomato-negative. Liver injury was induced in these mice by exposure to CCl4 twice per week for 12 weeks, after which they were treated with 5C or solvent control for four weeks. We observed little difference between the 5C treatment group and controls in the number of tdTomato-negative hepatocyte-like cells in liver sections from Alb-CreER: Rosa-tdTomato mice. The number of tdTomato-negative hepatocyte-like cells in Alb-CreER: Rosa-tdTomato mice (indicating hepatocyte-like cells transdifferentiated from NPCs) was approximately 12 times lower than tdTomato+ hepatocyte-like cells in Col1a2-CreER: Rosa-tdTomato mice (indicating hepatocyte-like cells induced from both transdifferentiation and revitalization), after 5C treatment during injury (Figures 4E and 4F). These results indicated that the transdifferentiation of NPCs to hepatocytes was negligible or very low efficiency, while 5C could induce the formation of hepatocyte-like cell colonies (indicated by Col1a2-traced) that mainly originated from Col1a2-traced hepatocytes rather than NPCs.

In addition, immunostaining indicated that Col1a2 traced CYP3A4^+^, and Hnf4a^+^ hepatocyte-like cells could only be detectable in the liver, but not brain, heart, lung, spleen, kidney, or muscle (Figure S8), further supported that the original cells of *in vivo* revitalization were dHeps but not mesenchymal cells residing in most of the organs.

### 5 compounds ameliorates liver fibrosis and restores liver function in chronic liver injury model mice *in vivo*

**Figure 5:**
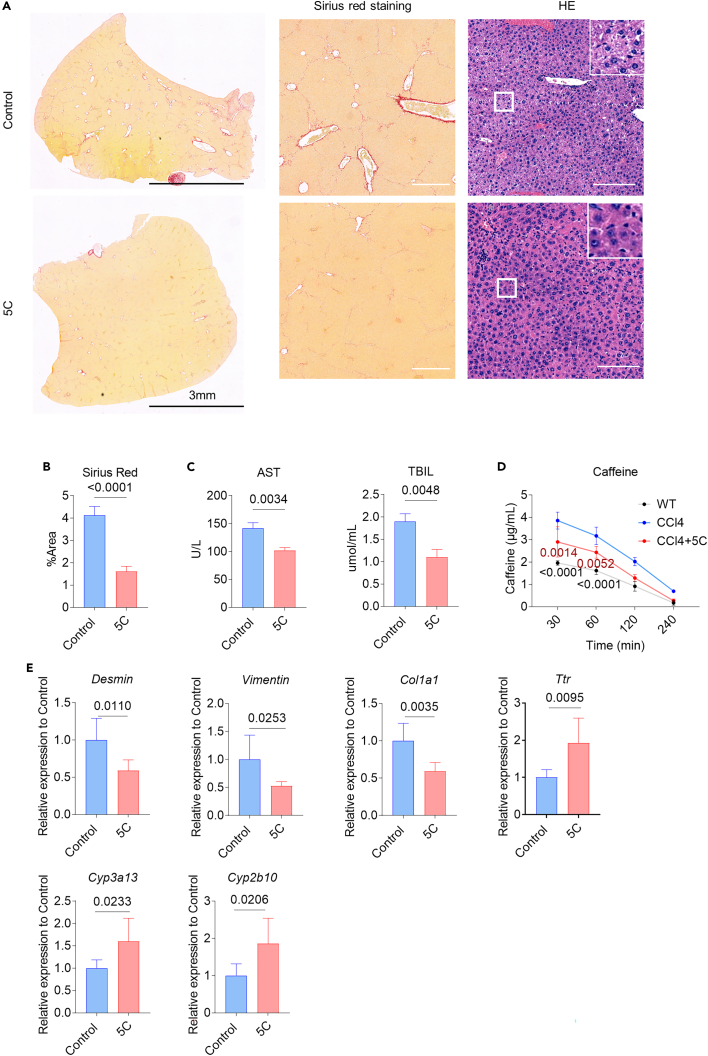
Inducing hepatocyte revitalization *in vivo* ameliorates liver fibrosis and restores liver function in a liver injury mouse model (A and B) H&E and Sirius red staining showed less fibrosis in 5C-treated mice in chronic liver fibrosis models than in controls. H&E, Scale bar, 200 μm, Sirius red staining, Scale bar, 400 μm. (C) Reduced levels of serum transaminases suggest an improved liver function in chemical-treated mice (n = 3) relative to controls (n = 3). Significance was assessed using an unpaired Student’s *t* test. (D) Pharmacokinetics of caffeine. Data represent mean ± SD. WT n = 3, CCl4 n = 3, CCl4+5C n = 3. WT Healthy mice, CCl4 solvent control mice after CCl4 injection, and CCl4+5C 5C-treated mice after CCl4 injection. Two-way ANOVA was performed to determine statistical significance. (E) Reduced levels of mRNA expression of myofibroblast and hepatocyte markers in 5C-treated mice compared to control mice. Significance was assessed using an unpaired Student’s *t* test.

We then examined whether the revitalization of injured hepatocytes *in vivo* could lead to functional recovery after liver injury. Sirius red staining revealed a 2.5-fold reduction in fibrotic area in 5C-treated mice (Figures 5A and 5B). Analysis of serum total bilirubin (TBIL) and aspartate aminotransferase (AST) also showed that these liver injury markers were reduced in 5C-treated mice (Figure 5C). In addition, pharmacokinetic analysis of caffeine (a Cyp1a2 probe drug) showed a significant hepatic function improvement in 5C-treated mice with CCl4-induced liver injury compared to non-model control mice, indicating the decrease in liver enzyme activity induced in CCl4 model could be significantly rescued by treatment with 5C (Figure 5D). These were also supported by the downregulated expression of myofibroblast markers (*Desmin*, *Vimentin*, and *Col1a1*) and upregulated expression of hepatocyte markers (*Cyp3a13*, *Cyp2b10*, and *Ttr*) in liver tissues by qPCR analysis (Figure 5E). We also assessed the efficacy of 5C in alleviating liver fibrosis in mice treated with DDC diet. Liver injury was induced in these mice by the dietary administration of DDC for 8 weeks, after which they were treated with 5C or solvent control for 4 weeks. Sirius red staining revealed a 1.85-fold reduction in fibrotic area in 5C-treated mice. AST was also reduced in 5C-treated mice compared with the solvent control (Figure S9). The findings demonstrate that *in vivo* hepatic revitalization induced by 5C could ameliorate liver fibrosis and promote functional recovery after chronic liver injury.

Additionally, we also tested the effect of 5C treatment in healthy mice to assess the potential side effects of 5C *in vivo*. No noticeable body weight loss or histological change of different organs was detected in 5C-treated mice compared with the solvent control for four weeks (Figure S10).

### GSK429286A, CID755673, and ETC-1002 (3C) were major contributors to hepatocyte revitalization

**Figure 6:**
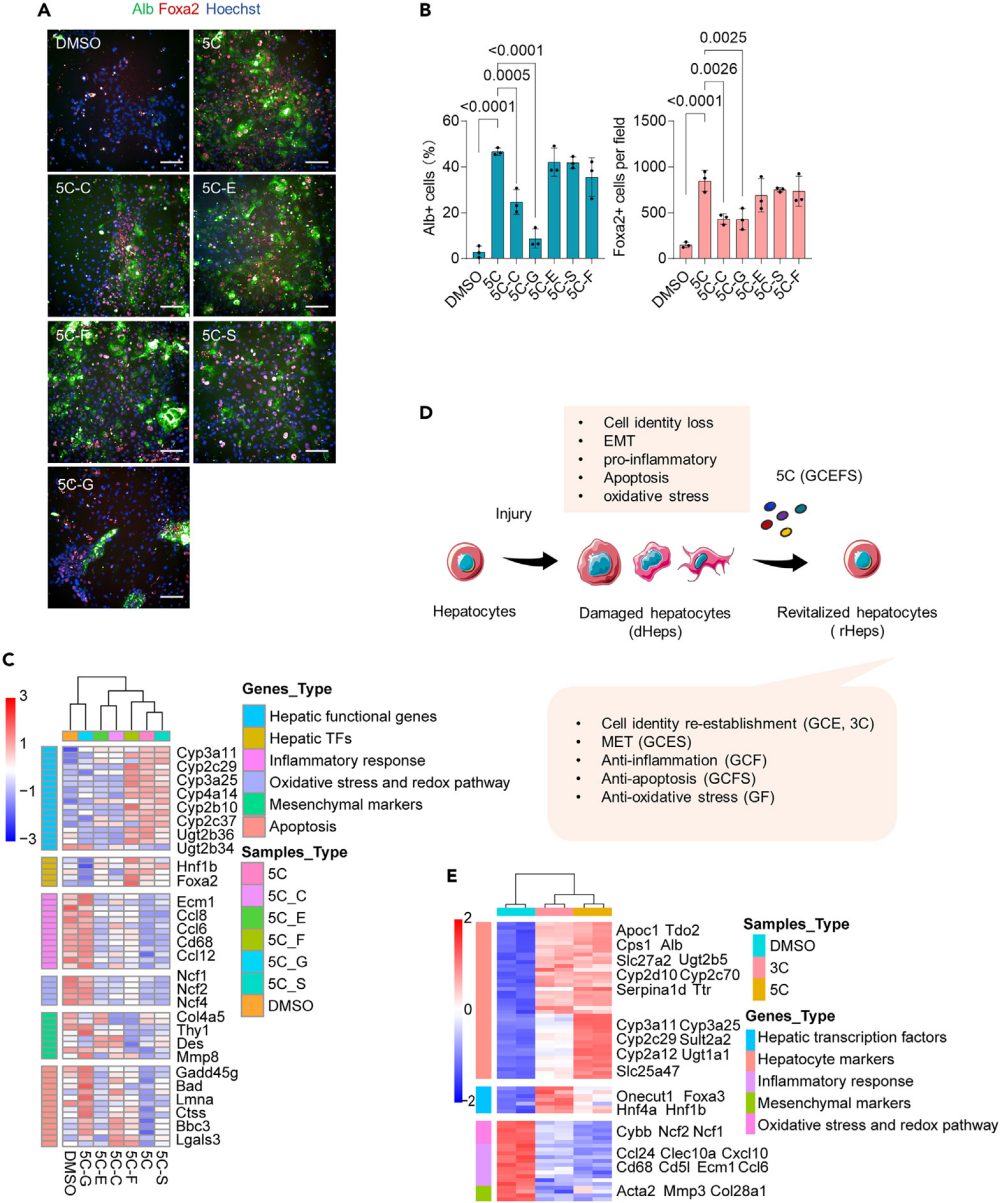
GSK429286A, CID755673, and ETC-1002 (3C) were major contributors to hepatocyte revitalization (A) The morphology of Alb-positive (green), and Foxa2-positive (red) of dHeps cultured in 5C with or without each component. GSK429286A, CID75567, ETC-1002, Salidroside, and Forskolin are represented in the diagram as G, C, E, S, and F, respectively. Scale bar, 100 μm. (B) The percentage of Alb-positive cells, and the number of Foxa2-positive cells hepatocytes after the treatment of 5C with or without each component in dHeps isolated from a CCl4-induced mouse model. Results are means ± SD for three biological replicates. Significance was assessed using one-way ANOVA. (C) Heatmaps showing the expression of genes associated with hepatic TFs, hepatocyte functional genes, inflammatory response, EMT, and oxidative stress and redox pathway to examine each chemical contributed to hepatocyte revitalization by removing each component of the 5C induction cocktail individually. The color bar indicates gene expression in Log2 scale. n = 2. (D) Schematic of each chemical contributed to hepatocyte revitalization. (E) Heatmaps showing the upregulation of genes associated with hepatic TFs, hepatocyte markers, and the downregulation of genes associated with inflammatory response, mesenchymal markers, and oxidative stress and redox pathway after the treatment of 3C, 5C compared with DMSO in dHeps isolated from CCl4-induced mouse model. Log2 fold-change>1. The color bar indicates gene expression in Log2 scale.

We next examined how each chemical contributed to hepatocyte revitalization by removing each component of the 5C induction cocktail individually. Immunostaining showed that removal of either GSK429286A or CID755673 led to lower proportions of Alb^+^ cells and Foxa2^+^ cell counts compared with the 5C treatment group (Figures 6A, 6B, and S11A). Further, analysis by qPCR and RNA-seq indicated that removal of GSK429286A, CID755673, or ETC-1002 resulted in a striking downregulation in the mRNA levels of hepatic TFs and functional genes, while the upregulation of fibrosis-associated factors (Figures 6C and S11B). Notably, removal of either GSK429286A or FSK upregulated the expression of inflammatory-, apoptosis-, and oxidative stress-related genes, and removal of CID755673 upregulated the expression of inflammatory-, and apoptosis-related genes, while removal of salidroside only upregulated the expression apoptosis-related genes (Figures 6C and 6D). These findings suggested that GSK429286A, CID755673, and ETC-1002 (3C) were essential to reverse the dHeps-associated transcriptional program.

We then compared whole transcriptome bulk RNA-seq data of dHeps isolated from CCl4-injured mice and treated with either 5C, 3C, or DMSO for 16 days. Notably, the expression of hepatocyte-enriched TFs (*Hnf6*, *Hnf4a,* and *Foxa3*) and hepatocyte functional genes (*Cyp3a11*, *Ugt1a1,* and *Cyp2a12)* was upregulated in both the 5C and 3C groups, while mesenchymal markers (*Acta2*, *Mmp3*), inflammatory-related genes (*Cxcl10*, *Ecm1*, *Ccl6*, and *Cd68*)*,* and oxidative stress-related genes (*Ncf2* and *Ncf1*), were down-regulated compared with their expression in the DMSO control group (Figures 6E and S11C). GO analysis indicated that differentially downregulated genes in the 3C group compared to the DMSO controls were enriched in “inflammation,” and “collagen metabolic process” terms, while upregulated genes were enriched in “epithelial cell proliferation” and “metabolic processes,” similar to terms enriched in the 5C-treated cells, further supporting that 3C was sufficient to initiate hepatocyte revitalization (Figure S11D). It was also noteworthy that the expression levels of hepatocyte-enriched TFs were higher in the 3C group than in 5C, while the expression of drug metabolism enzymes, such as *Cyp3a11*, *Cyp3a25,* and *Cyp2c29,* was lower in 3C group than 5C (Figure 6E). These results indicated that 3C was sufficient to restore the hepatocyte-specific transcriptional program in dHeps, and resulting in more extensive dedifferentiation than that induced by 5C.

### Reestablishment of transcription factor networks of damaged hepatocytes during hepatocyte revitalization induced by 3 compounds

**Figure 7:**
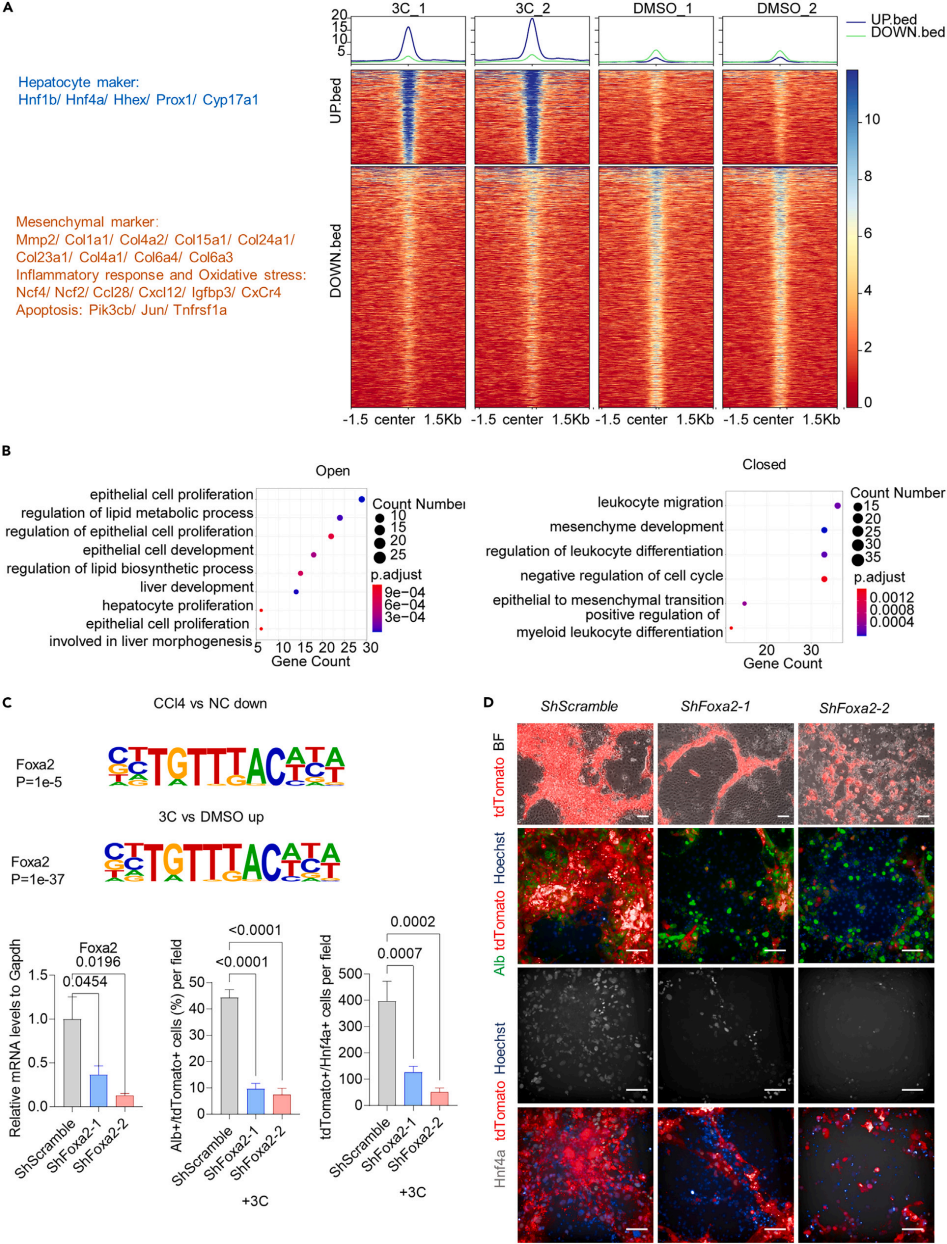
Reestablishment of transcription factor networks of dHeps during hepatocyte revitalization induced by 3C (A) ATAC-seq analysis of 3C and DMSO group (n = 2). Heatmap showed the landscapes of open (blue) and closed (yellow) peaks around peak center. Log2 fold-change>2, p value <0.05. (B) Gene Ontology (GO) analysis of genes located with open or closed chromatic loci in dHeps isolated from a CCl4-induced mouse model treated with control (DMSO) or 3C for 16 days. Log2 fold-change>1, p-adjust <0.05. (C) The percentage of Alb-positive epithelial cells, and the number of Hnf4a-positive cells labeled by AAV-TBG-Cre: Rosa-LSL-tdTomato hepatic cell tracing system after the transduction of *shFoxa2*, or scramble with 3C at day 16. Results are means ± SD for three biological replicates. Significance was assessed using one-way ANOVA. Relative mRNA levels of *Foxa2* were measured by qPCR after the transduction of shRNA. Results are means ± SD for two biological replicates. Significance was assessed using one-way ANOVA. (D) The morphology of Alb-positive epithelial cell colonies, and the number of Hnf4a-positive cells labeled by AAV-TBG-Cre: Rosa-LSL-tdTomato hepatic cell tracing system after the transduction of *shFoxa2*, or scramble with 3C at day 16. Scale bar, 100 μm.

To better understand the mechanism by which 3C induced revitalization, we next conducted epigenetic analysis of dHeps isolated from CCl4-induced mouse model collected after 16 days of DMSO or 3C treatment to identify their differences in transcriptional regulation. Assay for targeting accessible-chromatin with high-throughout sequencing (ATAC-seq) data revealed that hepatocyte-enriched TFs, such as *Hnf4a, Hnf1b*, *Hhex,* and *Prox1*, and genes associated with epithelial cell proliferation and liver development were located in loci with open chromatin, whereas markers associated with mesenchymal transition, cell-cycle arrest, apoptosis and inflammation (e.g., *Col1a1*, *Mmp2*, *Ccl28, CxCr4, Ncf2*) were located in closed chromatin loci in the 3C group (Figures 7A, 7B, and S12A).

To expand our understanding of the mechanisms by which 3C could induce chromatin-level changes in TF regulation, we performed motif analysis on open chromatin loci in the 3C group for comparison with that in DMSO control dHeps. We also conducted motif analysis for closed chromatin loci in hepatocytes from mice with CCl4-induced liver injury for comparison with those from healthy liver (NC). We found that binding motifs for hepatocyte-enriched TFs, including Foxa1, Foxa2, Hnf1b, Hnf6, Hnf6b, Foxa3, Pxr, and Hnf4a, were highly enriched in open loci of the 3C group as well as in closed loci of the CCl4-injured hepatocytes (Figure S12B), with the most significant enrichment detected in Foxa1 and Foxa2 DNA binding motifs in open loci of the 3C group.

Foxa2 is one of the hepatocyte lineage-determining transcription factors,^12^^,^^25^ and immunofluorescence staining showed that the proportions of Foxa2^+^ hepatocyte-like cells was restored by chemical compounds during hepatic revitalization (Figures 1E and 1F). To determine whether Foxa2 was involved in hepatic revitalization induced by chemical compounds, we then knocked down *Foxa2* in dHeps in the presence of 3C. Interestingly, knockdown of *Foxa2* resulted in the dramatic suppression of 3C-induced Alb expression, Hnf4a^+^ cell counts, and cell viability (Figures 7C and 7D). These data indicated that the 3C cocktail could revitalize dHeps by reactivating hepatocyte-enriched TFs, involving Foxa2.

### Chemical induction of hepatocyte revitalization in injured human hepatocytes

We subsequently explored whether 3C could also induce revitalization in human hepatocytes. We here designed an *in vitro* model of human hepatocyte injury induced by TGF-β, which was reported to induce EMT and apoptosis in human hepatocytes.^26^ The injured hepatocytes cannot efficiently self-recover after TGF-β withdraw. In contrast, the treatment of 3C cocktail in these injured hepatocytes for 9 days, resulted in the amelioration of cell death, as indicated by the increased percentage of ALB^+^ cells in comparison with DMSO control (Figures S13A–S13C).

Subsequent RNA-seq analysis in cells collected after 3 days of DMSO or 3C treatment revealed global transcriptomic changes under 3C treatment compared with that in control cells (Figure S13D). In particular, genes involved in bile secretion, cell cycle, cell population proliferation, and metabolic pathways (including *SLC51A*, *OTC*, *UGT2B7*, *BUB1B*, *CCNB2*, *MKI67*, and so forth) were upregulated under 3C treatment, suggesting the effect of 3C on hepatocytes proliferation and regeneration. The differentially downregulated genes under 3C treatment were associated with apoptosis, EMT, necroptosis, and TNF signaling (including *VIM*, *TGFB1*, *MDK*, *ECM1*, *SMAD3*, *MAP2K3*, *FOS*, *CXCL2*, and so forth) (Figures S13E and S13F). GO analysis showed that the genes that were upregulated in the 3C group compared with the DMSO control cells were enriched for the terms “nuclear division,” “chromosome segregation,” “cytokinesis,” and “cell cycle checkpoint signaling,” while the genes that were downregulated were enriched for the terms “extracellular matrix organization,” and “response to reactive oxygen” (Figure S14). These data indicated that the 3C cocktail could revitalize human hepatocytes displaying apoptotic and EMT phenotypes induced by TGF-β, and promote hepatocyte proliferation and maintenance.

## Discussion

Our study demonstrates a chemically induced revitalization process for injured hepatocytes that can promote liver repair in chronic mouse models of liver injury. This *in vivo* chemically induced revitalization strategy provides several clear advantages over cell lineage reprogramming (i.e., transdifferentiation). First, this method is technically easier to implement, which is necessary for clinical translation. Although hepatocyte identity is partially lost in dHeps, these cells still maintain basal expression levels of some liver-enriched TFs as well as epigenetic marks from healthy hepatocytes, which facilitates their reprogramming compared with changes required to reprogram other lineages. Second, chemically induced revitalization raises fewer concerns about intermediate cell types, because all “intermediates” in the cell revitalization process are, in principle, healthier than the original, injured phenotype.

Recent studies have shown that resetting transcription factor networks that are disrupted in degenerative disease (e.g., phenobarbital and CCl4-induced liver injury) can correct a cirrhotic phenotype in hepatocytes. For example, re-expression of *HNF4A* was shown to inhibit fibrosis in four independent mouse models of liver fibrosis.^27^ Moreover, Foxa2 overexpression in the liver can reportedly alleviate hepatic fibrosis.^28^ These studies suggest that the re-establishment of a dysregulated hepatocyte-specific transcription factor network to reprogram the injured hepatocyte phenotype in chronic hepatic failure represents a promising approach for liver regeneration. Interestingly, we found that 3C could reverse injury-associated changes in epigenetic level, and this reversal was particularly striking among hepatocyte-enriched TFs such as *Foxa2*, *Hnf4a,* and *Foxa3*. These findings indicate that chemically induced revitalization also involves rebuilding transcription factor networks disrupted during liver injury, and resembles a partial reprogramming approach.

Moreover, we observed that dHeps were more sensitive to 5C treatment in cell expansion than healthy hepatocytes in *in vitro* TGF-β-induced hepatocyte injury model. Considering the differences in selectivity for hepatocyte growth and function between *in vitro* and *in vivo* conditions, we also examined whether 5C could induce dHep revitalization *in vivo* using a Col1a2 inducible tracing system, since injured hepatocytes tend to express EMT-associated genes such as Col1a2, as shown in our scRNA-seq data (Figure S1). Lineage tracing based on the activation of Col1a2 promoter in uninjured controls (Figure S6) showed that the Col1a2 inducible tdTomato signal could not label healthy hepatocytes without injury, but only labeled hepatocytes in the CCL4 model mice, indicating that the Col1a2 promoter was strictly responsive to injury in this lineage tracing system, thereby precluding the possibility of apparent leaky expression by healthy hepatocytes. Furthermore, the number of Col1a2-traced cells increased by > 10-fold after treatment with 5C (Figures 4C and 4D), while the total liver weight was not dramatically changed compared with controls (Figure 3E). These results indicated the vast majority of healthy hepatocytes in the CCl4 liver injury model were not significantly expanded, even if there was some potential leakage of Col1a2-induced labeling of healthy hepatocytes. Cumulatively, Col1a2 tracing showed that 5C treatment more selectively expands dHeps *in vivo*, although we cannot strictly exclude the possibility that 5C could also affect healthy hepatocytes, which warrants closer scrutiny in future studies.

In this study, we observed that fibrosis was apparently resolved, and liver function was significantly improved following treatment with 5C. These beneficial effects may be due to the restoration of hepatocyte identity and function in dHeps, as well as the inhibition of cellular senescence. Hepatocellular senescence has been proposed to decrease hepatic regeneration in models of severe liver damage, including extended hepatectomy,^29^ acetaminophen treatment,^30^ and cirrhosis.^31^ In our current work, whole transcriptome analysis and senescence-associated β-galactosidase activity assays showed that the chemicals used to induce hepatic revitalization also lead to escape from cellular senescence.

This study also brought to light new questions that require further investigation. In particular, it remains unclear whether a chemically induced revitalization strategy can be extended to facilitate regeneration in other organs. Furthermore, our future work will necessarily examine whether chemicals identified in our study can also rejuvenate cells to reverse aging-associated phenotypes, just like the partial reprogramming with Yamanaka factors,^32^^,^^33^^,^^34^^,^^35^^,^^36^ since these chemicals can restore cell function and suppress cell senescence. Overall, this study illustrates a promising strategy for regenerative medicine through the chemically induced revitalization of injured cells to repair tissues and resolve fibrosis.

### Limitations of the study

Col1a2-CreER: Rosa26-tdTomato mice were used to label Col1a2-traced dHeps and Col1a2-traced liver myofibroblasts after CCl4-induced injury *in vivo*. However, this tracing system cannot comprehensively describe the severity of damage in dHeps *in vivo*, since dHep populations could be heterogeneous. Although we show that this tracing system based on transgenic *Col1a2* promoter activation cannot label healthy hepatocytes *in vivo* without injury, we cannot exclude the possibility that some healthier hepatocytes may be labeled after injury *in vivo*. In addition, the efficiency of CreER-mediated recombination is not 100%, and the state of Col1a2-traced dHeps requires detailed characterization in future work.

## STAR★Methods

### Key resources table

**Table:** 

REAGENT or RESOURCE	SOURCE	IDENTIFIER
**Antibodies**		

goat anti-Alb	Bethyl	Cat#A90-134A; RRID:AB_2891982
goat anti-Hnf4a	ThermoFisher Scientific	Cat#MA5-14891; RRID:AB_10980013
rabbit anti-Foxa2	Abcam	Cat#ab108422; RRID:AB_11157157
Rabbit anti-CYP3A4	Proteintech	Cat#18227-1-AP; RRID:AB_2090329
Rabbit anti-Ki67	Abcam	Cat#Ab15580; RRID: AB_443209
goat anti-Human ALB	Bethyl	Cat#A80-129A; RRID:AB_2891968
Rabbit anti-Desmin antibody	Abcam	Cat#ab32362; RRID:AB_731901
Goat anti-type I collagen	Southern biotech	Cat#131001; RRID:AB_609609
Donkey anti-Rabbit IgG (H + L) Highly Cross-Adsorbed Secondary Antibody, Alexa Fluor Plus 555	ThermoFisher Scientific	Cat#A32794; RRID:AB_2762834
Donkey anti-Rabbit IgG (H + L) Highly Cross-Adsorbed Secondary Antibody, Alexa Fluor Plus 488	ThermoFisher Scientific	Cat#A32790; RRID:AB_2762833
Alexa Fluor™ 647 donkey anti-rabbit IgG (H + L)	invitrogen	Cat#A31573; RRID: AB_2536183
Alexa Fluor™ 647 donkey anti-goat IgG (H + L)	invitrogen	Cat#A21447; RRID: AB_141844
Alexa Fluor™ 488 donkey anti-goat IgG (H + L)	invitrogen	Cat#A11055; RRID: AB_2534102
anti- RFP (tdTom)	Chromotek	Cat#5F8
CD45 MicroBeads	Milyenti Biotec	Cat#130-052-301; RRID:AB_2877061

**Bacterial and virus strains**		

AAV.TBG.PI.Cre.rBG	addgene	Cat#107787

**Biological samples**		

Cryopreserved human hepatocytes	BioreclamationIVT	Cat#M00995-P

**Chemicals, peptides, and recombinant proteins**		

Recombinant Human EGF	PeproTech	Cat#AF-100-15
Recombinant Human HGF	PeproTech	Cat#100-39H
TGF-β1	PeproTech	Cat#96-100-21-10
ETC-1002	Selleck	Cat#S7953
GSK429286A	Selleck	Cat#S1474
Forskolin	Selleck	Cat#S2449
Salidroside	Targetmol	Cat#T2717
CID755673	Selleck	Cat#S7188
Nicotinamide	Selleck	Cat#S1899
Dexamethasone	Sigma-Aldrich	Cat#D4902
SB431542	Selleck	Cat#S1067
DAPT	Selleck	Cat#S2215
IWP2	Selleck	Cat#S7085
LDN193189	Selleck	Cat#S2618

**Critical commercial assays**		

Periodic-Acid-Schiff	Solarbio	Cat#G1360
Senescence Associated β-galactosidase Staining	Beyotime	Cat#C0602
Oil Red O Stain Kit	Solarbio	Cat#G1262
CellTracker™ Green CMFDA	ThermoFisher	Cat#C2925
Periodic-Acid-Schiff	Solarbio	Cat#G1360
TransScript® One-Step gDNA Removal and cDNA; Synthesis SuperMix	TransGen	Cat#AT311
ChamQ SYBR qPCR Master Mix	Vazyme	Cat#Q321-02
TruePrep DNA library Prep Kit V2 for Illumina	Vazyme	Cat#TD501

**Deposited data**		

RNA-seq	This paper	GSE233081; GSE233082; GSE233083
scRNA-seq	This paper	GSE233084
ATAC-seq data	This paper	GSE233080

**Experimental models: Organisms/strains**		

Mouse: Alb-CreER mice	Professor Bin Zhou	N/A
Mouse: Col1a2-CreER mice	Professor Bin Zhou	N/A
Mouse: Rosa26-tdTomato mice	Professor Bin Zhou	N/A

**Oligonucleotides**		

Foxa2 shRNA targeting sequence: CCGCAAGATTTC ACCAGCATT	This paper	N/A
Foxa2 shRNA targeting sequence: CGAGCACCATT ACGCCTTCAA	This paper	N/A
Scramble shRNA targeting sequence: GTCTCCACG CGCAGTACATTT	This paper	N/A

**Recombinant DNA**		

pLKO.1-Foxa2-1	This paper	N/A
pLKO.1-Foxa2-2	This paper	N/A
pLKO.1-Scramble	This paper	N/A

**Software and algorithms**		

Prism 9.0	GraphPad Software, Inc.	https://www.graphpad.com/scientificsoftware/prism/
Harmony	PerkinElmer	N/A
Zen	Zeiss	N/A
Seurat	https://satijalab.org/seurat/	N/A
ImageJ	National Institutes of Health, USA	https://imagej.nih.gov/ij/
DESeq2 1.36.0	DESeq2	https://bioconductor.org/packages/release/bioc/html/DESeq2.html
R package v4.0.4	R Project	https://www.rstudio.com/products/rpackages/
The Integrative Genomics Viewer	http://software.broadinstitute.org/software/igv/download	N/A

**Other**		

GluMAx	Gibco	Cat#35050-061
Polybrene	Macgene	Cat#MC032
MgCl_2_·6H_2_O	Sigma	Cat#M2670
KCl	Sigma	Cat#P5405
Na_2_HPO_4_	Sigma	Cat#S5136
NaH_2_PO_4_	Sigma	Cat#S3139
HEPES	Sigma	Cat#H4034
Glucose	Sigma	Cat#G7021
CaCl_2_·2H_2_O	Sigma	Cat#C7902
Tris-HCl	Thermo fisher	Cat#15568025
EGTA	Sigma	Cat#67-42-5
DNaseI	Sigma-Aldrich	Cat#D5025
Tamoxifen	Sigma	Cat#10540-29
Salt-T4® DNA Ligase	NEB	Cat#M0467S
*Age* I-HF	NEB	Cat#R3552S
*EcoR* I-HF	NEB	Cat#R3101T
DDC	Research Diets, inc.	Cat#D111122201
CCl4	Rhawnseal	Cat#RH416503
PEG300	Sigma-Aldrich	Cat#8074841000
Tween 80	Sigma-Aldrich	Cat#P4780
Corn oil	Acros	Cat#405435000
HCM Bulletkit	Lonza	Cat#CC3198
Pronase	Roche	Cat#10165921001
Collagenase P	Roche	Cat#11213873001
Williams Medium E	Gibco	Cat#A12176-01
Gibco™ Collagen I, rat tail	Gibco	Cat#A1048301
B-27™ Serum-Free Supplement (50×)	Thermo fisher	Cat#A3582801
DMSO	Sigma	Cat#D2650
Normal donkey serum	Jackson	Cat#017-000-121
DH5α	TsingkeBiotechnology	Cat#T-TSC-C14
Penicillin-streptomycin	Thermo Fisher	Cat#15140-122
Fetal Bovine Serum (FBS)	VISTECH	Cat#SE100-011

### Resource availability

#### Lead contact

Further information and requests for resources and reagents should be directed to and will be fulfilled by the lead contact, Yang Zhao (yangzhao@pku.edu.cn).

#### Materials availability

This study did not generate new unique reagents.

#### Data and code availability

Single-cell RNA-seq data, RNA-seq data, and ATAC-seq have been deposited at GEO and are publicly available as of the date of publication. Accession numbers are listed in the key resources table.

This paper does not report original code.

Any additional information required to reanalyze the data reported in this work paper is available from the lead contact upon request.

### Experimental model and study participant details

#### Mice ethical approval

All procedures involving mice were approved by the Institutional Animal Care and Use Committee at Peking University, Beijing (IMM-ZhaoY-2), and Nanjing Jingruikang Molecular Pharmaceutical Technology Co., Ltd, Nanjing (IACUC-2021-025). We used 8–10 weeks old Rosa26-tdTomato mice, Alb-CreER: Rosa26-tdTomato mice, and Col1a2-CreER: Rosa26-tdTomato mice for lineage-tracing experiments. The Rosa26-tdTomato mice, Alb-CreER mice, and Col1a2-CreER mice were kindly provided by Professor Bin Zhou (Institute of Biochemistry and Cell Biology, Chinese Academy of Sciences). Alb-CreER: Rosa26-tdTomato mice were generated by crossing heterozygous Alb-CreER mice with heterozygous Rosa26-tdTomato mice. Col1a2-CreER: Rosa26-tdTomato mice were generated by crossing heterozygous Col1a2-CreER mice with heterozygous Rosa26-tdTomato mice. All mice are C57BL/6 background.

#### Cell lines

##### Cryopreserved human hepatocytes

We used cryopreserved human hepatocytes from male humans provided by BioreclamationIVT (Product number: M00995-P, Male Human hepatocytes). The cryopreserved primary human hepatocytes were resuspended in OptiPlate Hepatocyte Media (Shanghai Quan Yang Co., Ltd). Cell viability was verified by Trypan blue (Gibco) and seeded in a collagen-I-coated dish at 1 × 105 cells/well in a 24-well plate. The cells were then cultured at 37°C, using Normoxia (5% CO2 incubator). The medium was changed to X5C medium^16^ 4 h after seeding and every 3 days thereafter.

### Method details

#### Primary cell isolation

##### Digestion protocol #1 (whole liver cells isolation)

Mouse liver from CCl4-injured mice was washed with phosphate buffered saline (PBS). The liver was minced with a scalpel, digested in 2.5 mg/ml Collagenase II, 2.5 mg/ml Collagenase IV and 0.1 mg/ml DNase I at 37°C for 1 h with agitation, and then strained through a 40 μM nybolt mesh. The cell suspension was centrifuged at 800g for 5 min, supernatant removed, and cell pellet resuspended in red blood cell lysis buffer for 5 min. Then the cell suspension was again centrifuged at 800g for 5 min. The supernatant was removed, cell pellet was resuspended in high glucose Dulbecco’s modified Eagle’s medium (DMEM) supplemented with 10% fetal bovine serum (FBS) and 1% penicillin-streptomycin.

##### Digestion protocol #2 (hepatocyte isolation)

Mouse primary hepatocytes from CCl4-injured mice were isolated using a two-step, *in situ* collagenase perfusion method. The hepatic portal vein was cannulated *in situ*, perfused with EGTA, Pronase (7mg per mouse), and Collagenase (1mg per mouse), and liver specimens were further digested by Pronase/Collagenase. After perfusion, filtered cell suspensions were harvested. The hepatocytes were seeded on rat tail collagen-coated plates and changed to serum-free Hepatocyte Maintenance Medium (Lonza) after seeding for 5h.

#### Chemical screen

Mouse whole liver cells were isolated from mice with CCl4-induced chronic liver fibrosis (protocol #1). The cells were transferred to a hepatocyte culture medium with chemical compounds after passage; this culture medium has previously been used to induce hepatocytes from mouse fibroblasts.^12^ After our modifications, it consisted of DMEM/F-12 supplemented with 10% FBS, 1% ITS (insulin-transferrin-selenium) premix, 1 μM dexamethasone, 10 mM nicotinamide, 1% GlutaMAX, 1% penicillin-streptomycin (Invitrogen), 20 ng/mL hepatocyte growth factor (Peprotech), and 20 ng/mL epidermal growth factor (Peprotech). The medium was changed every 3 days. The chemical libraries were arrayed in a 96-well plate, which consisted of the kinase library (MCE Kinase inhibitor library, L1600-Kinase Inhibitor Library), an epigenetic library (L1200-Epigenetic Inhibitor Library, MCE Epigenetic Inhibitor Library), and an approved drug library (MCE Approved Drug Library, L1000-Approved Drug Library, and Drug repurposing library-Selleck). The final working concentration of the compounds was 2μM. After 16 days, the cells were tested for protein expression of Alb via immunostaining. We measured the number of Alb-positive colonies, and candidate compound hits were determined as those that had 2-fold greater effects on the induction of Alb-positive colonies than the negative control.

#### Chemical induction of hepatocyte revitalization in human and mouse hepatic cells

Injured mouse hepatocytes were isolated from CCl4-injured mice (protocol #2) with either the 5C cocktail (i.e., Salidroside 5 μM, CID755673 50 μM, Forskolin 2 μM, GSK429286A 2 μM, and ETC-1002 5 μM), a 3C cocktail (i.e., CID755673 50 μM, GSK429286A 2 μM, and ETC-1002 5 μM), or the solvent control (DMSO) in serum-free Hepatocyte Maintenance Medium (Lonza). After 16 days, the cells were tested for protein expression or harvested for mRNA isolation preparation. For TGF-β-induced injury in mouse primary hepatocytes, mouse hepatocytes were induced by TGF-β (5 ng/mL) for 24h, after which they were treated with 5C or DMSO in serum-free Hepatocyte Maintenance Medium (Lonza) for 3–5 days. On day 3, the cells were tested for growth curve of albumin and Foxa2 double-positive hepatocytes, and hepatocytes were collected for function and polarization of hepatocytes on day 5.

Cryopreserved human hepatocytes were induced by a high dose (50 ng/mL) of TGF-β for 5 days, after which they were treated with a 3C cocktail or DMSO in X5C hepatocyte maintenance medium^16^ for 3–9 days. On day 3, the cells were collected for RNA-seq analysis and tested for protein expression on day 9.

#### Animal models

To induce liver fibrosis, 8–10 weeks old Rosa26-tdTomato mice, Alb-CreER: Rosa26-tdTomato mice, and Col1a2-CreER: Rosa26-tdTomato mice (both male and female) received intraperitoneal injections and were injected with 2.5 μL/g of 10% CCl4 (Sigma), dissolved in corn oil twice per week for 12 weeks. To induce cholestatic liver fibrosis, Col1a2-CreER: Rosa26-tdTomato mice (both male and female) were fed with a DDC (Research Diets, inc. D111122201) diet for 8 weeks. In this study, a mixture of male and female mice was used experimentally. No sex differences were noted.

#### AAV virus infection and tamoxifen administration

The plasmid of AAV-TBG-Cre, adeno-associated virus (AAV) serotype 8 particles expressing Cre recombinase driven by liver-specific thyroxine-binding globulin (TBG) was purchased from Addgene. Virus packaging was provided by Lianyungang Chuangrei Biological Products Trading Co., Ltd. To specifically label hepatocytes, 8–10 weeks old Rosa-tdTomato mice were intravenously injected at 2 × 10^11^ viral genome copies per mouse, followed by a washout period of 2 weeks. Tdtomato was expressed in >99.5% of hepatocytes 2 weeks after injection. The mice were used as injury models after AAV injection. For low-density lineage tracing, Rosa-tdTomato mice were intravenously injected at 5.58 × 10^7^ viral genomes per gram body weight (vg/g), followed by a washout period of 2 weeks.

To label liver Col1a2-traced cells, tamoxifen was fed to 8–10 weeks old Col1a2-CreER: Rosa-mice were fed at a dose of 4mg/30g body weight 3 times during chronic liver fibrosis. To label hepatocytes, tamoxifen was fed to 8–10 weeks old Alb-CreER: Rosa-mice at a dose of 4mg/30g body weight 3 times, followed by a 2- weeks washout period before inducing chronic liver fibrosis.

#### Delivery of 5C or the solvents control into mice after inducing fibrosis

After inducing fibrosis, AAV-TBG-Cre: Rosa26-tdTomato mice, Alb-CreER: Rosa26-tdTomato mice, and Col1a2-CreER: Rosa26-tdTomato mice were systemically treated with 5C for 4 weeks while control mice were treated in the same manner but with solvents instead of chemical compounds. We injected 4.5 mg/kg/day CID755673, 8.6 mg/kg/day GSK429286A, and 8.2 mg/kg/day Forskolin in 6.8% DMSO and PEG/T (30% PEG300 (Sigma), 5% Tween80 (Sigma), 65% double distilled water), and 4 mg/kg/day salidroside in PBS intraperitoneally, and the mice orally received 20 mg/kg/day ETC1002 in Carboxymethyl cellulose and Tween 20 with a final pH 7–8, as previously reported^37^ at the same time after liver fibrosis.

#### Immunofluorescence

Cells were washed with PBS and fixed in 4% paraformaldehyde (PFA) for 15 min at room temperature. After washing twice with PBS, the cells were permeabilized and blocked in PBS containing 0.2% Triton X-100 and 3% donkey serum for 1 h at room temperature. Then the cells were incubated with primary antibodies at 4°C overnight. After washing three times with PBS, they were incubated with secondary antibodies at 37°C for 1 h. The nuclei were stained with Hoechst (Yeasen) for 5 min. The secondary antibodies were donkey anti-rabbit IgG (H + L) Highly Cross-Adsorbed Secondary Antibody, Alexa Fluor 488, Alexa Fluor 647, and Alexa Fluor 555.

Cryosection staining was performed by dissecting the liver samples in PBS, followed by 4-8h fixation in 4% PFA (Sigma), overnight dehydration in 30% sucrose at 4°C, embedding in O.C.T., and freezing in liquid nitrogen. A 10-μm frozen section was prepared (Leica CM1950) for antibody staining, which was stored at −20°C unless it was immediately stained. It was then incubated in TBS for 20 min at 37°C, in blocking buffer (10% normal donkey serum, 2% BSA, and 0.1% Triton X-100 in PBS) for 1h at room temperature, and then in antibody buffer overnight at 4°C. Afterward, sections were washed 3 times for 5 min each in PBS, hybridized with antibodies Alexa Fluor 488 or Alexa Fluor 555 (Thermo Fisher) for 1h in 2% BSA-included PBS, washed 3 more times for 5 min each in PBS, and mounted with DAPI-Fluoromount-G (SouthernBiotech). Photos were taken via microscope (Zeiss LSM 710 confocal, Zeiss fluorescence microscope, and PerkinElmer Opera Phenix/Harmony system).

#### qPCR

Total RNA was extracted using an EasyPure RNA Kit (TransGen Biotech) and was reverse-transcribed into cDNA using TransScript One-step gDNA Removal and cDNA Synthesis SuperMix (TransGen Biotech). Real-time PCR was performed on a Quantagene q225 System (Kubo Technology) using ChamQ SYBR qPCR Master Mix (Vazyme, Cat#Q321-02).

#### Histology and immunohistochemistry

Liver tissue was fixed with 4% PFA at 4°C overnight, embedded in paraffin, and cut at 5 μm for histological and immunohistochemical analysis. For histopathological examination, the sections were stained with hematoxylin-eosin (H&E). Sirius Red staining was used to determine collagen deposition. To generate cryosections, samples were fixed in 4% PFA at 4°C overnight and cryoprotected in 30% sucrose at 4°C overnight before embedding and freezing in O.C.T. Regarding tdTomato staining, 4-μm sections were incubated with blocking buffer (endogenous peroxidase) for 10 min, blocked with goat serum (ZSGB-bio, ZLI-9022) for 1h, stained with anti- RFP (tdTom) overnight (Chromotek, 5F8, 1:300), developed color with HRP goat anti-rat (Servicebio, GB23302) and DAB (ZSGB-bio, ZLI-9019), and finally counterstained with hematoxylin. Images were obtained with a Leica Aperio VERSA system.

#### Single-cell RNA-seq

Individual cells were isolated from the liver tissue of CCl4-injured mice and uninjured mice for single-cell RNA sequencing using 10X chromium workflow. Liver cells were isolated from Col1a2-CreER: Rosa26tdTomato mice, as previously described.^38^ After perfusion with EGTA, Pronase (14 mg per mouse), and Collagenase (3.7 U per mouse), liver specimens were further digested by Pronase/Collagenase and purified via Nycodenz density gradient centrifugation. The isolated cells were mixed with CD45 MicroBeads (Milyenti Biotec, 130-052-301) for 15 min at room temperature to remove the majority of CD45^+^ cells and collect CD45^−^ cells for further analysis.

#### Single-cell RNA-seq data alignment and quantification

Raw reads were processed to generate gene expression profiles using an in-house pipeline. Briefly, after filtering all read one sequences lacking a poly T tail, cell barcodes, and UMI were extracted. Adapters and poly-A tails were trimmed (fastp V1) before aligning read two to GRCh38 with ensemble version 92 gene annotation (fastp 2.5.3a and feature Counts 1.6.2). Reads with the same cell barcode, UMI, and gene were grouped to calculate the number of UMIs per gene per cell. The UMI count tables of each cellular barcode were used for further analysis.

#### Dimensionality reduction, clustering analysis

We performed unsupervised clustering and differential gene expression analyses in the Seurat R package v4.0.4. In particular, we used SNN graph-based clustering, where the SNN graph was constructed using from 2 to 10 principal components as determined by dataset variability shown in principle components analysis (PCA); the resolution parameter to determine the resulting number of clusters was also tuned accordingly. All UMAP visualizations were produced using Seurat functions in conjunction with the ggplot2.

#### *In vivo* analysis of CYP activity

Mice were dosed by oral gavage with probe drug caffeine (5 mg/kg). Whole blood (30μL–50μL) was obtained from the submandibular vein at intervals of drug post-administration (30, 60, 120, and 240 min) into a tube coated with heparin. Plasma samples were obtained by centrifugation for 10 min at 2000 rpm.

The plasma samples were processed with a standard protein precipitation method using methanol with propranolol as the internal standard. The supernatant was analyzed by a Shimadzu LCMS-8060 system (Shimadzu, Japan). The samples were eluted from a Kromasil 100-5C18 column (2.1 × 50 mm, 5 μm) using a mobile phase composed of water with 0.1% formic acid (A) and methanol (B) gradient. Analysis time was 5 min for a sample size of 10 μL with a flow rate of 0.3 mL/min. The samples were measured in positive ion mode while caffeine was quantified with the peak area of the extracted positive ion 195.1–138.2 m/z. The quantification method was validated as the standard curve ranged from 0.2 to 50 μg/mL. All the data were processed by non-compartmental analysis using the Phoenix WinNonlin (Certara, USA), as previously described, and used to quantify particular products by liquid chromatography-tandem mass spectrometry (LC-MS/MS).

#### RNA-seq

The pooled RNA-seq experiments were performed by Novogene (Nanjing, Chinese). Total RNA was extracted using the EasyPure RNA Kit (TransGen), then sequenced on NovaSeq6000-PE150. The reads were processed and mapped to the Mus musculus genome GRCm39 or the Homo Sapiens genome GRCh38. For heatmaps, FPKM was transferred as log2 (FPKM+1) and normalized over samples. Detection of the differentially expressed genes (DEG calling) between samples was performed with DESeq^39^ when samples included multiple repetitions. Only P-value <0.05, and Log2 fold change < −0.5/-1 or >1/0.5 were regarded as DEGs and used in further analysis. The front-rank DEGs were enriched for GO analysis.

#### ATAC-seq

ATAC-seq libraries were prepared using TruePrep DNA library Prep Kit V2 for Illumina (vazyme). Totally, 5 × 104 cells were used for every single reaction. Cells were washed in cold PBS and resuspended in 50 μL lysis buffer (10 mM Tris-HCl pH 7.4, 10 mM NaCl, 3 mM MgCl2, 0.5% NP-40) for 10 min, and nuclei were spun at 500 g for 10 min using a refrigerated centrifuge. Then, the pellet was resuspended in 50 μL transposase reaction mix and incubated at 37°C for 30 min. The samples were purified and purified, and then libraries were amplified by PCR for 14 cycles. The libraries were sequenced using an Illumina HiSeq 2,500 machine.

ATAC-seq analysis includes peak calling with MACS^40^ (version 2.1.2), differential peak detection with RPKM, and visualization with EnrichedHeatmap. Coverage tracks of the samples were generated with the alignment of reads (BAM file) with the bamCoverage function from deepTools (Ramirez et al., 2014). The number of reads per bin was calculated and normalized by reads per kilobase per million mapped reads (RPKM).

#### shRNA transfection

##### Construction of sh*Foxa2* lentiviruses

shRNAs targeting *Foxa2* or scramble sequence were co-transfected with psPAX2, pMD2.G into 293T cells using PEI reagent (Polysciences, 23966-1) for lentivirus production. Viruses were collected from cell culture supernatant and used to infect dHeps isolated from CCl4-injured mice (protocol #2) with Polybrene (1000×) (Macgene, MC032). Puromycin (Invivogen, ant-pr-1) was used for one week to select successfully infected cells. The knockdown efficiency of shRNAs was verified by RT-PCR.

#### Assays for PAS, CMFDA, senescence-associated β-galactosidase, and oil red staining

Cells with different treatments at day 16 were stained by Periodic-Acid-Schiff (Solarbio, G1360), and oil red staining kit (Solarbio, G1262) according to the manufacturer’s instructions. For senescence-associated β-galactosidase staining, the cells with different treatments at day 16 were fixed with 4% paraformaldehyde for 15 min at room temperature. Cells were stained by Senescence Associated β-galactosidase Staining (Beyotime) was performed according to the manufacturer’s instructions. For functional polarization assay, hepatocytes were incubated for 30 min with 2μM 5-chloromethylfluorescein diacetate (CMFDA) (ThermoFisher, C2925) and 1 μg/ml Hoechst 33342. Cultures were subsequently washed with PBS and imaging was performed on PerkinElmer Opera Phenix/Harmony system.

### Quantification and statistical analysis

Data are expressed as means ± SD. The value of n is mentioned in the figure/figure legends and always stands for separate biological replicates. The significance of difference between two groups was determined using an unpaired Student’s *t* test, with two-tailed p value. Comparison analysis for two parameters from two different groups was performed by two-way ANOVA or one-way ANOVA. A p value of <0.05 was considered significant. Prism 9.0 (Graphpad) was used for all analyses.
