# Supplementary material for: Chemically induced revitalization of damaged hepatocytes for regenerative liver repair
Source: iScience. 2023 Nov 23;26(12):108532. doi: 10.1016/j.isci.2023.108532 (PMC10746372; doi:10.1016/j.isci.2023.108532)
Supplement: Document S1. Figures S1–S14 and Table S1 [file mmc1.pdf]

iScience, Volume *26*

Supplemental information

Chemically induced revitalization of damaged hepatocytes for regenerative liver repair

Pengyan Lin, Yunfei Bai, Xinxin Nian, Jun Chi, Tianzhe Chen, Jing Zhang, Wenpeng Zhang, Bin Zhou, Yang Liu, and Yang Zhao

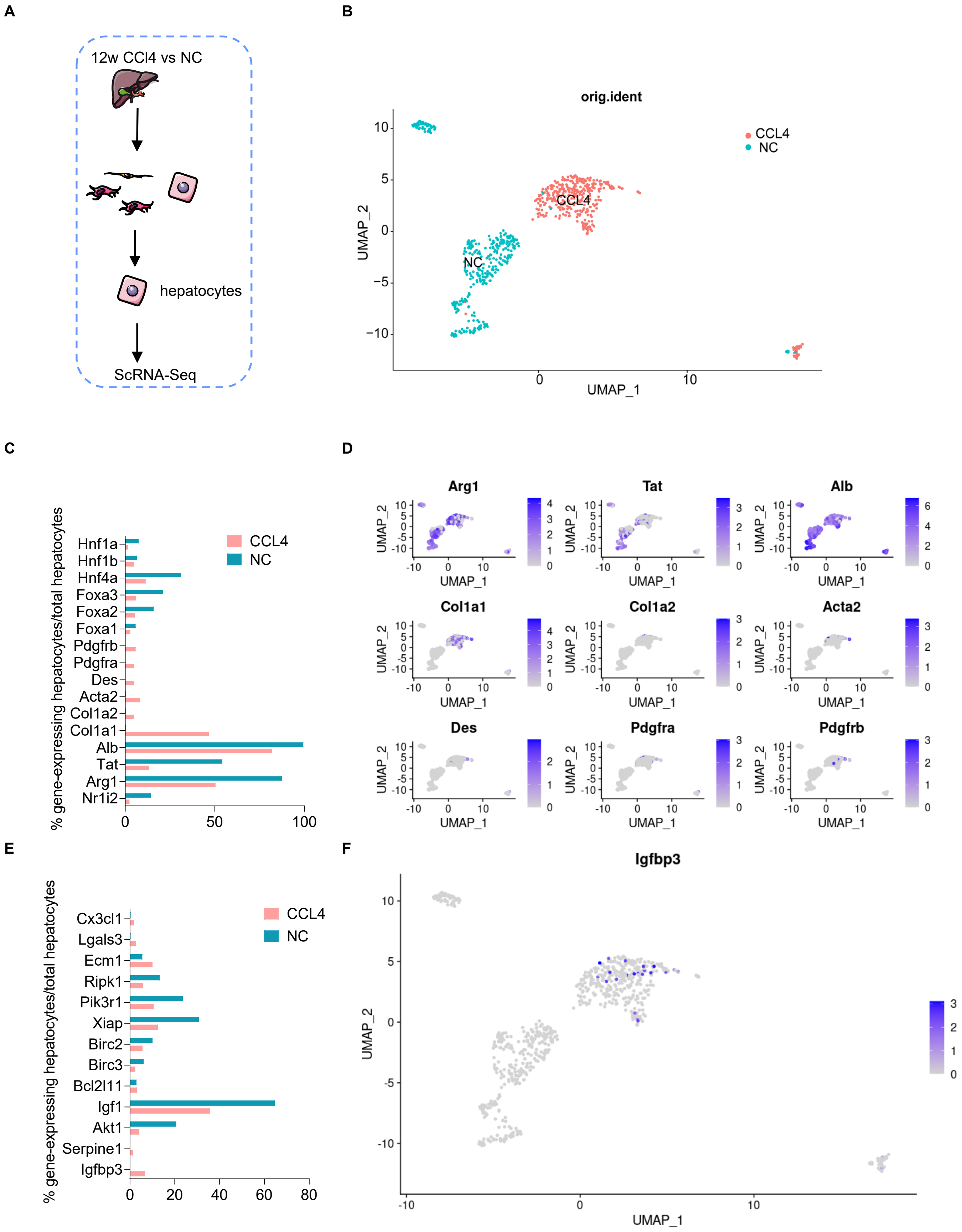

Figure S1. ScRNA-Seq analysis of hepatocytes isolated from healthy and CCl4-injured mouse livers，related to Figure 1

A

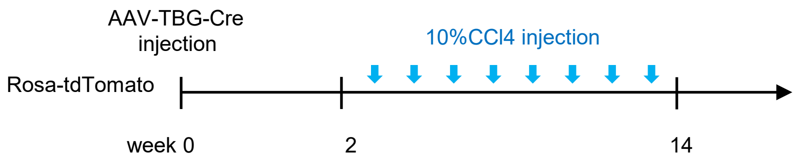

B

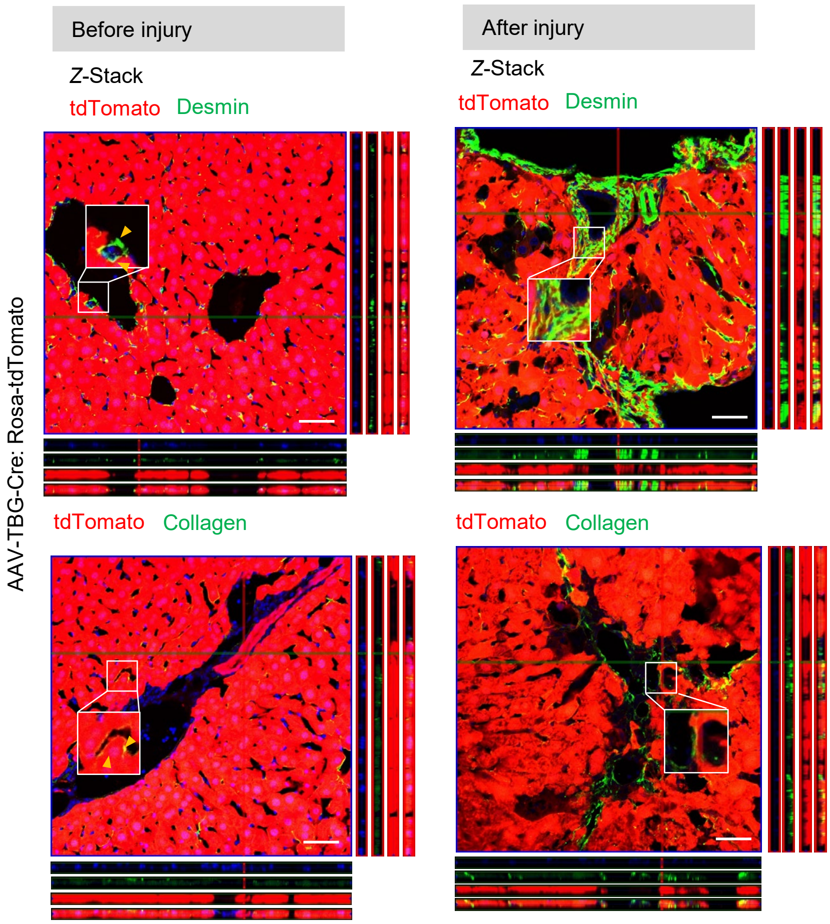

Figure S2. Characterization of dHeps from CCl4-induced mouse model by confocal analysis, related to Figure 1

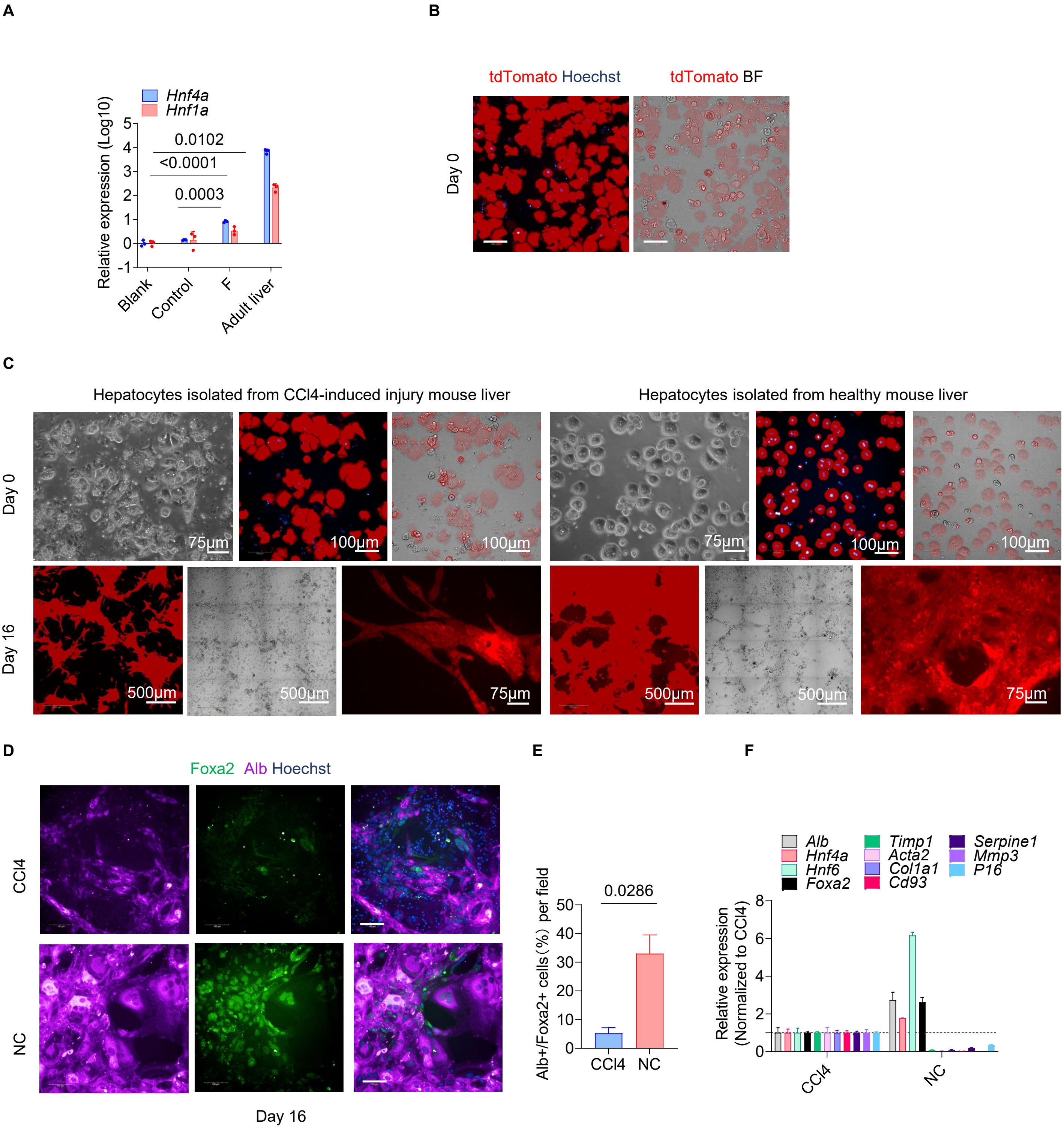

Figure S3. Characteristic comparison of isolated dHeps and healthy hepatocytes upon culture, related to Figure 1

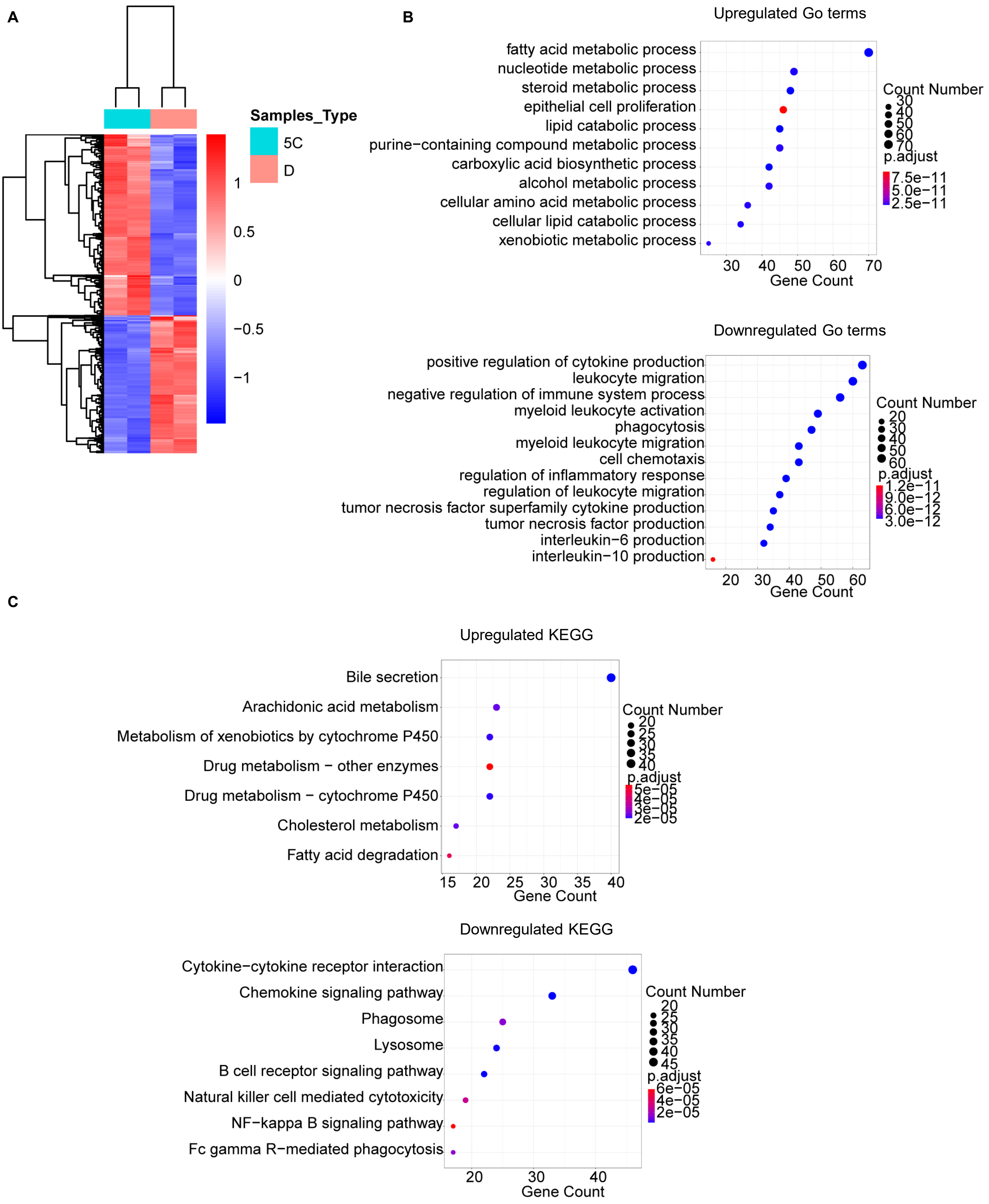

Figure S4. Gene expression analysis of dHeps isolated CCl4-induced mouse model treated with DMSO (D) or 5C for 16 days, related to Figure 1

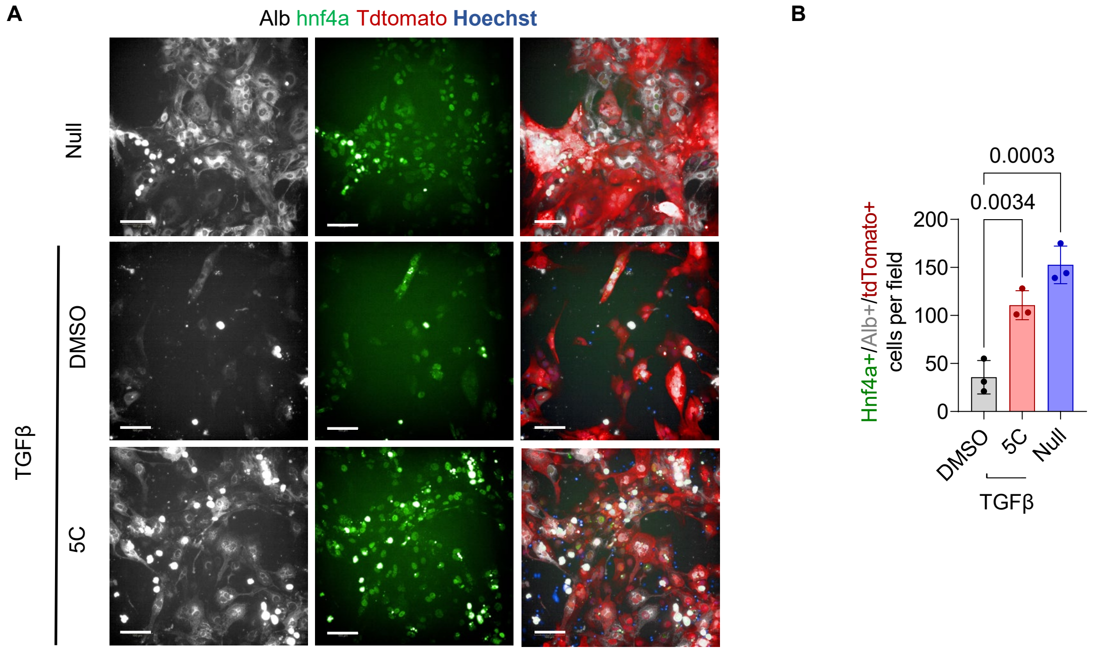

Figure S5. 5C induced the revitalization of hepatocytes injured by transforming growth factor β (TGF-β) treatment, related to Figure 2

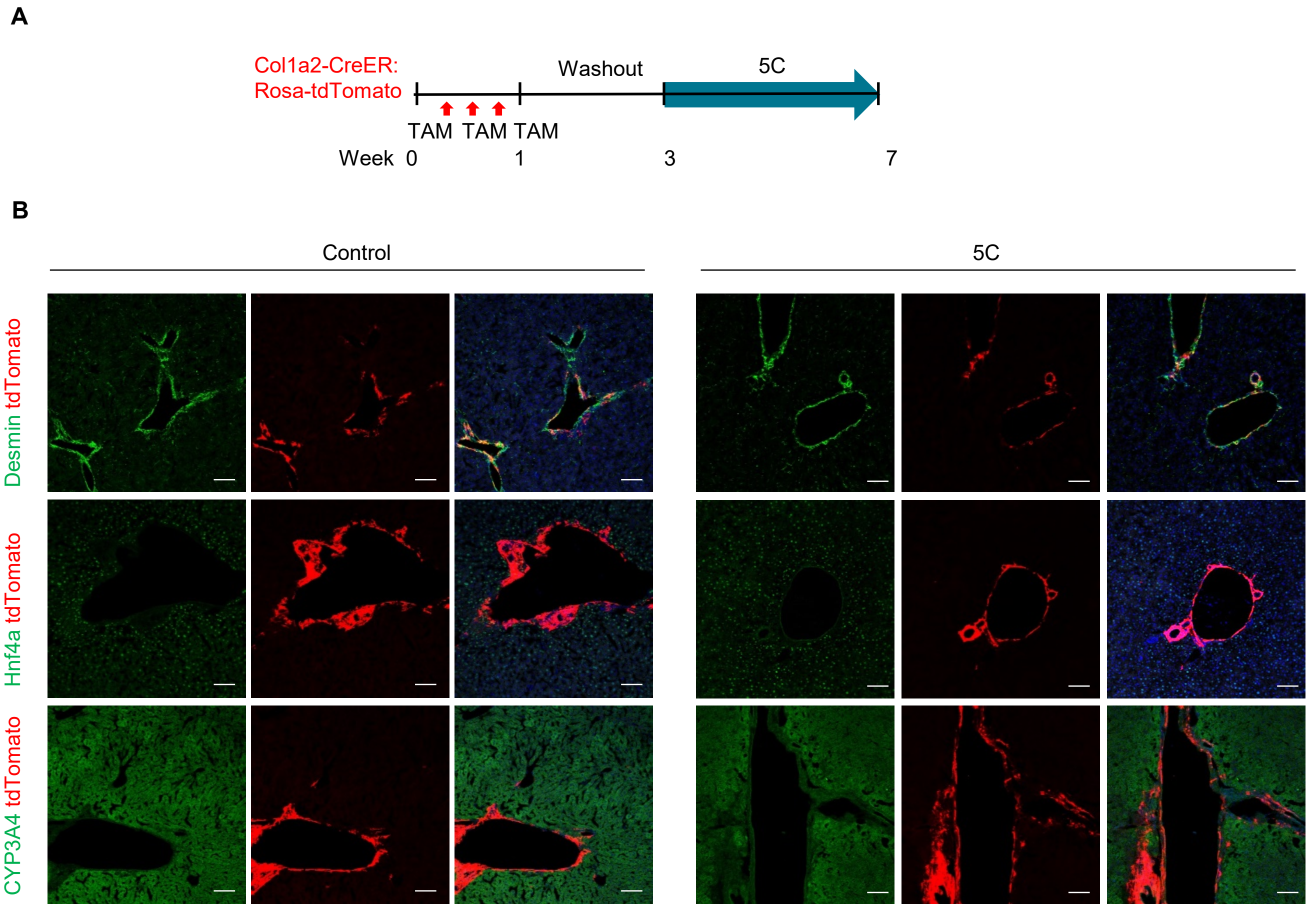

Figure S6. Characterization of Col1a2-CreER: Rosa-tdTomato labeling in mesenchymal cells after 5C treatment in healthy mice, related to Figure 4

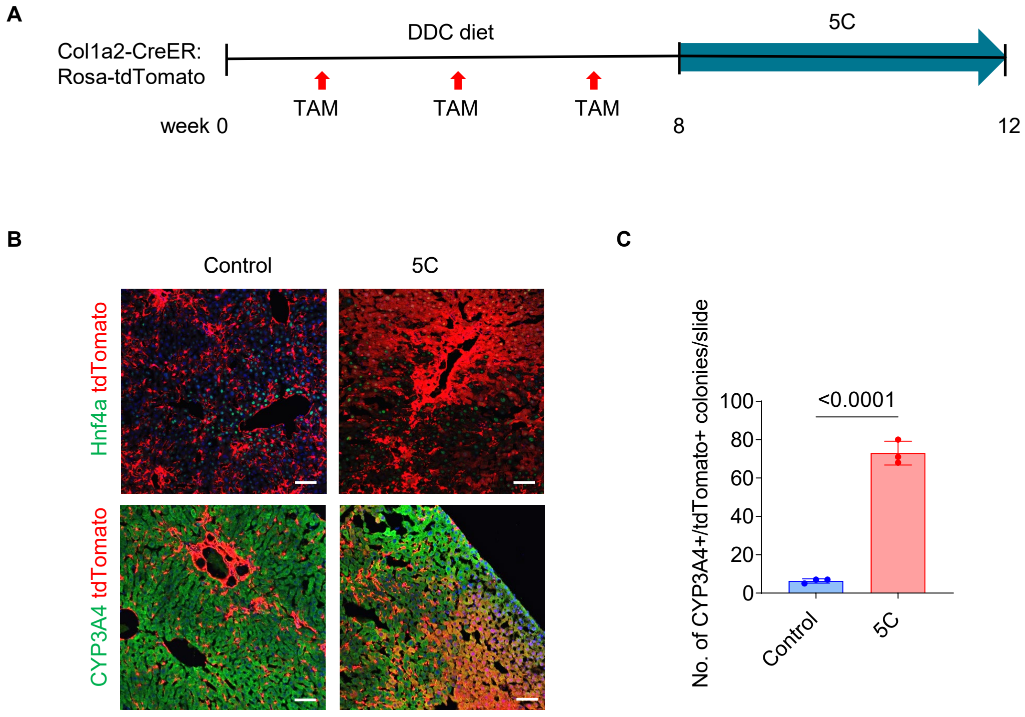

Figure S7. Restoration of regenerative potential in Col1a2-traced dHeps of mice with DDC-induced liver injury, related to Figure 4

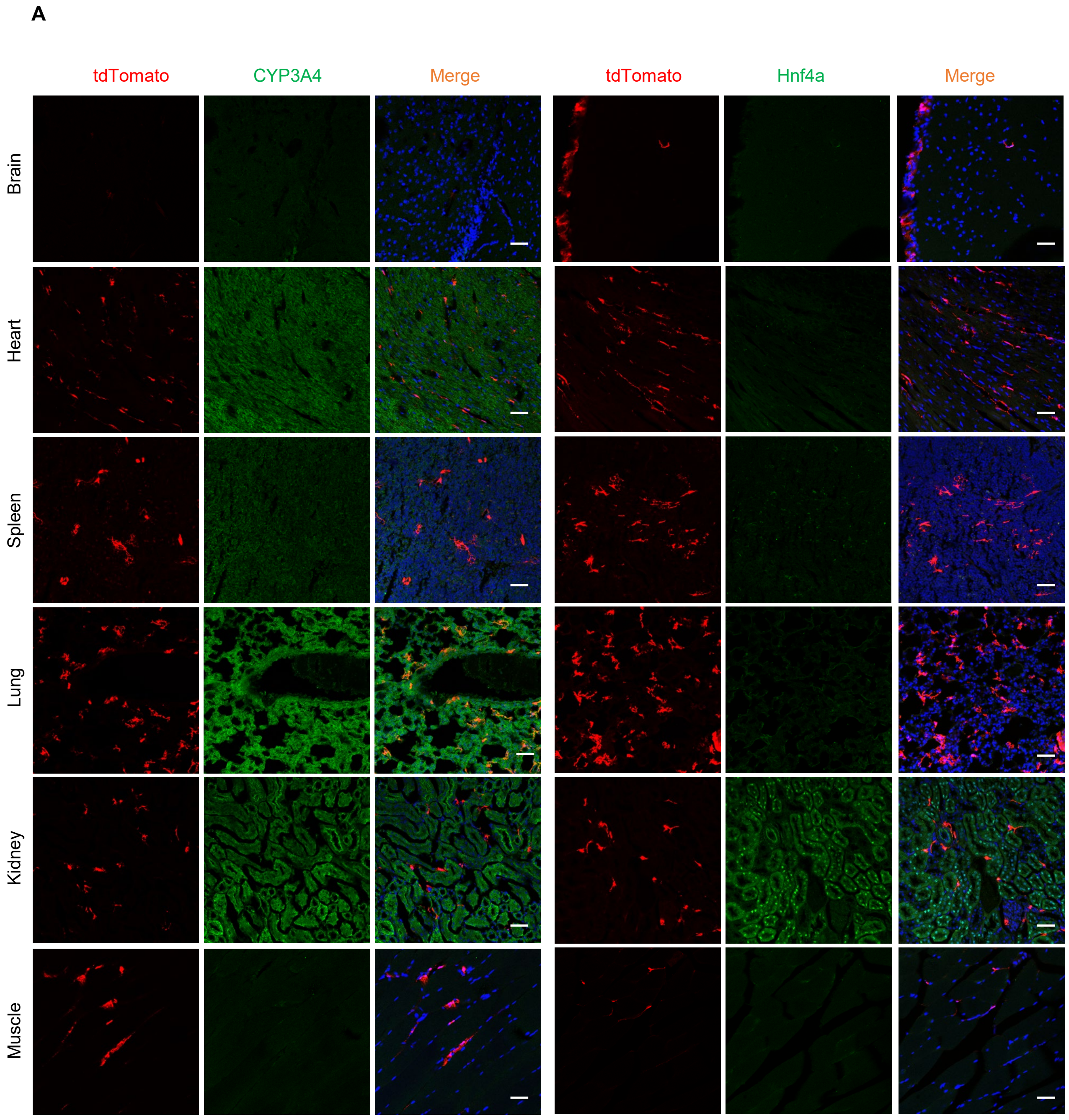

Figure S8. Absence of hepatocyte-like cells in organs other than the liver, related to Figure 4

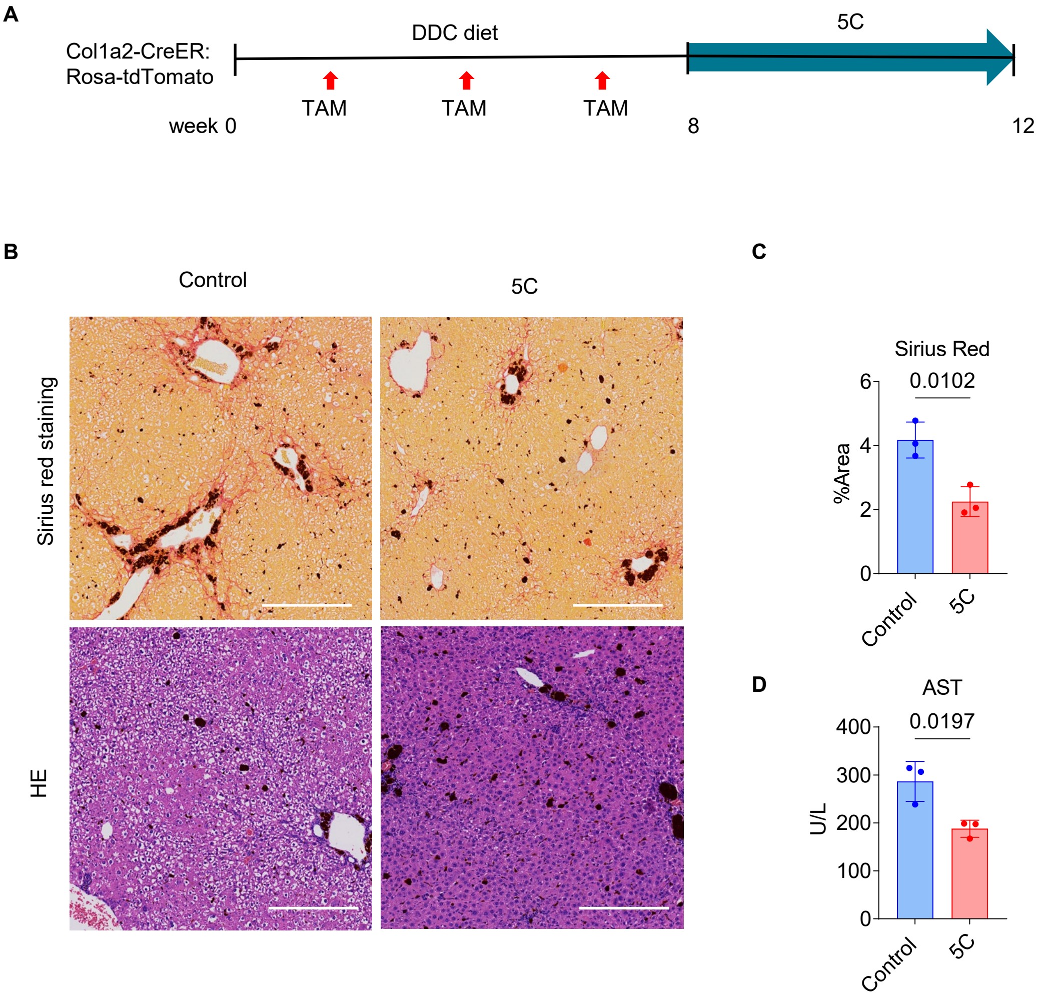

Figure S9. Effect of 5C treatment on liver fibrosis in mice with DDC-induced liver injury mouse model, related to Figure 5

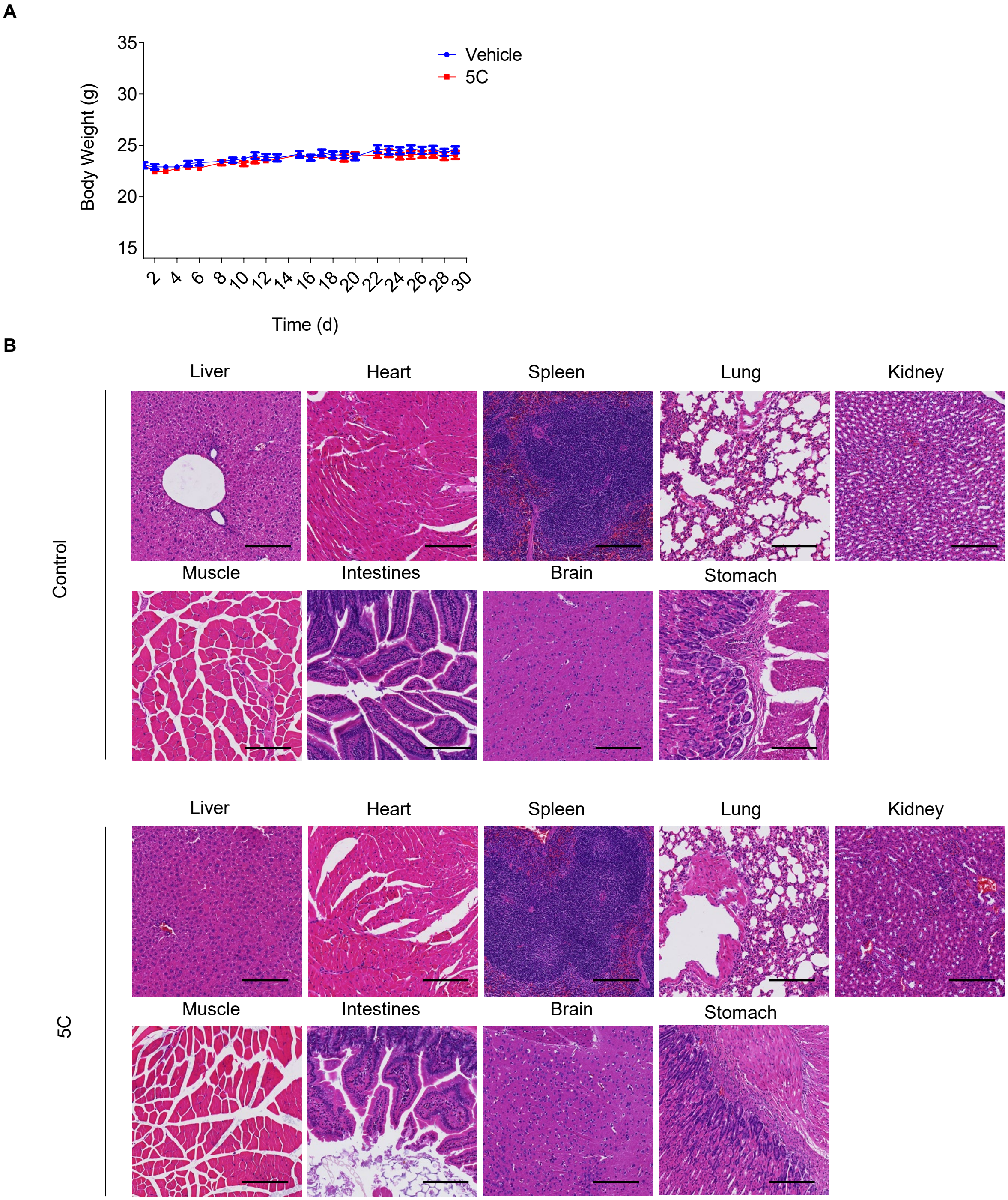

Figure S10. The effect of 5C treatment in healthy mice, related to Figure 5

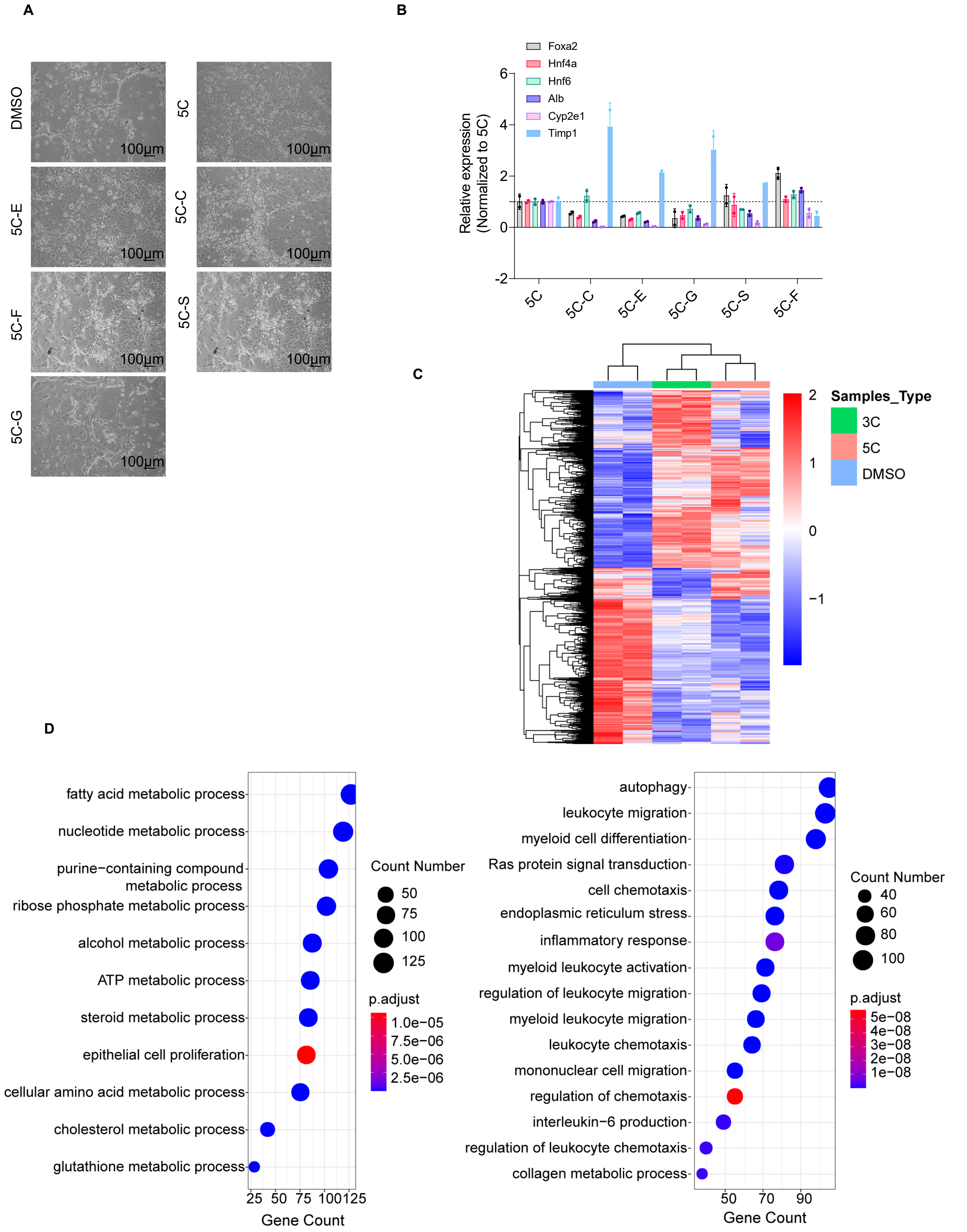

Figure S11. GSK429286A, CID755673, and ETC-1002 (3C) were major contributors to hepatocyte revitalization, related to Figure 6

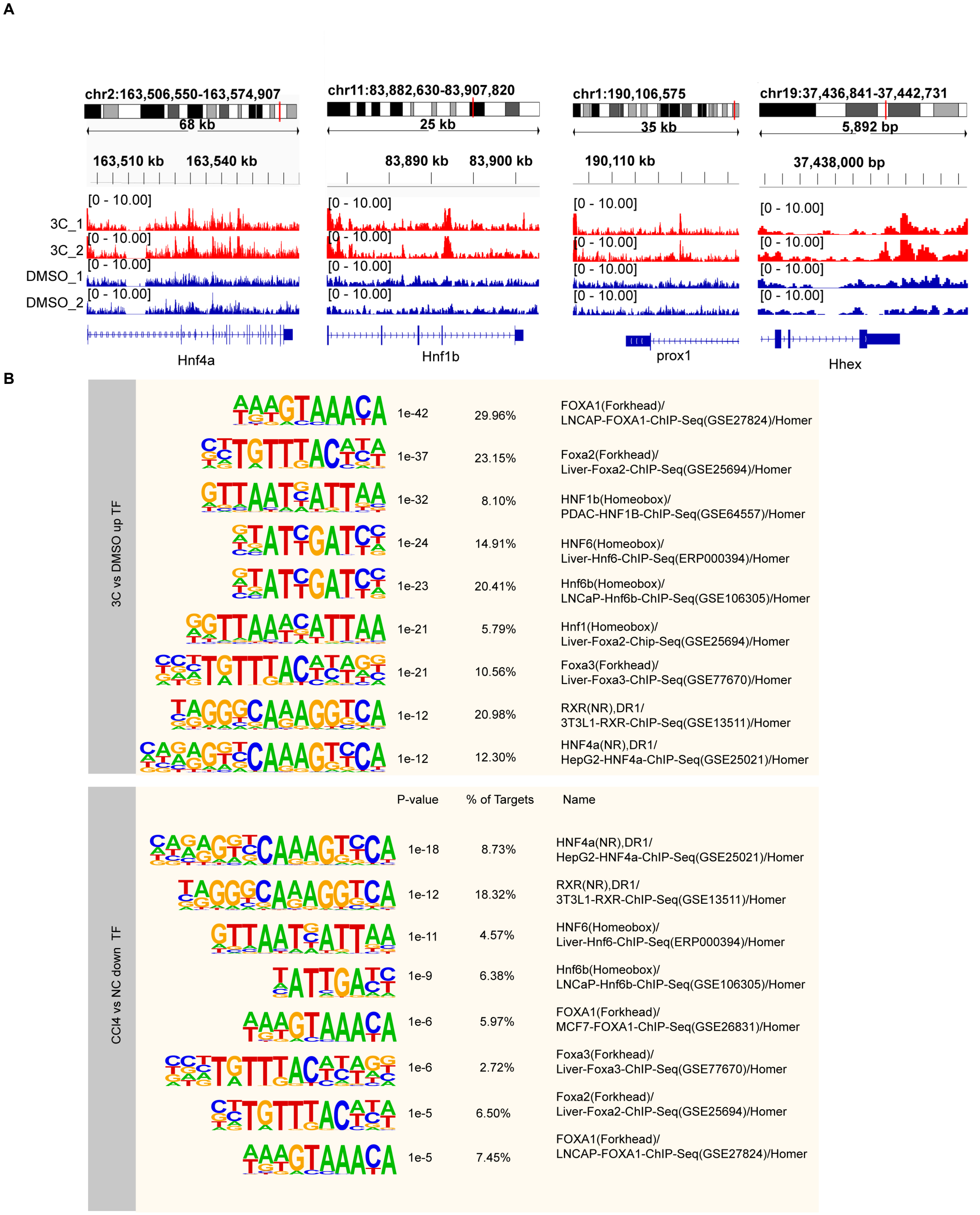

Figure S12. Chromatin accessibility analyses of chemical compounds to revitalize dHeps with identity loss, related to Figure 7

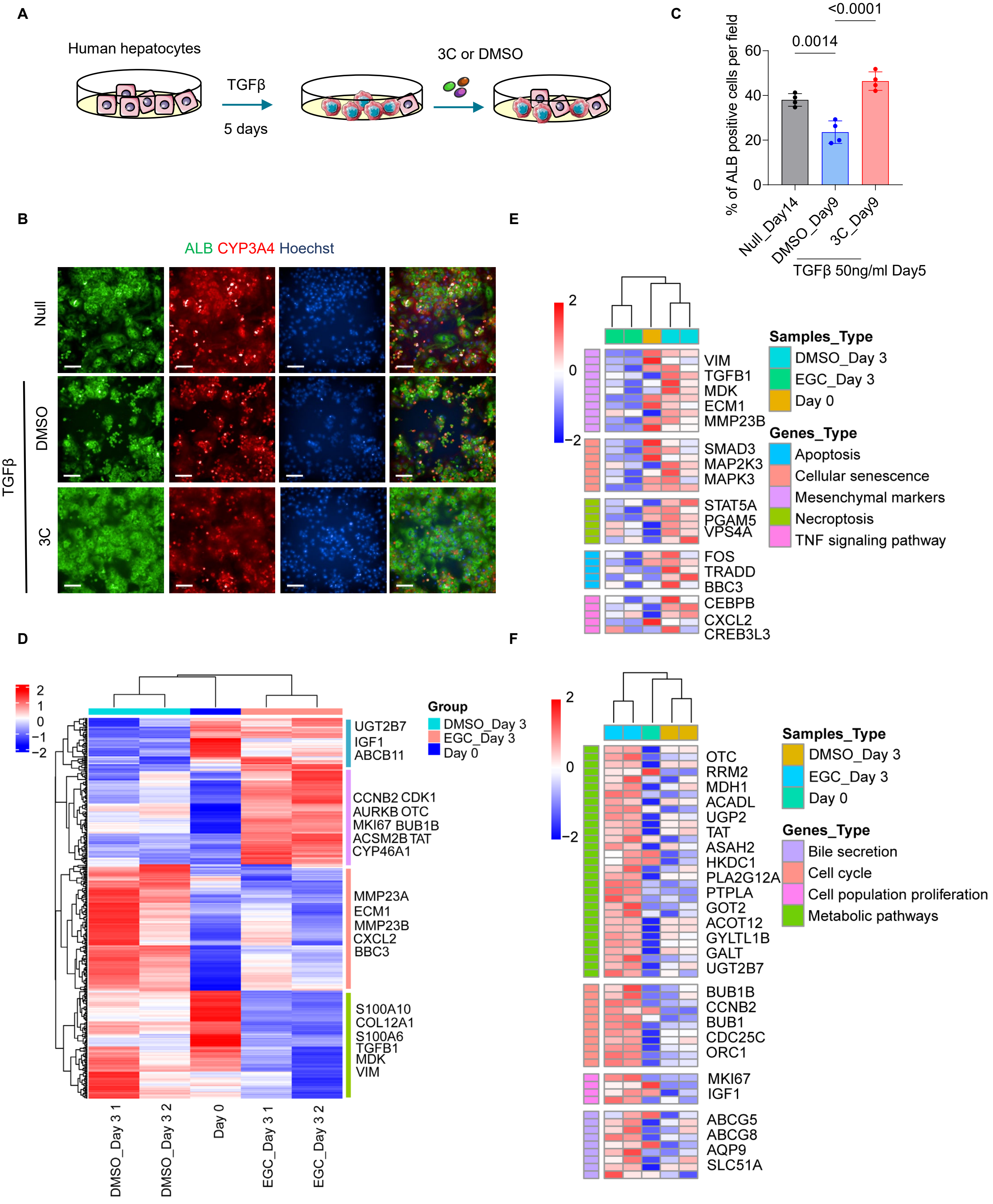

Figure S13. Chemical induction of hepatocyte revitalization in injured human hepatocytes

A

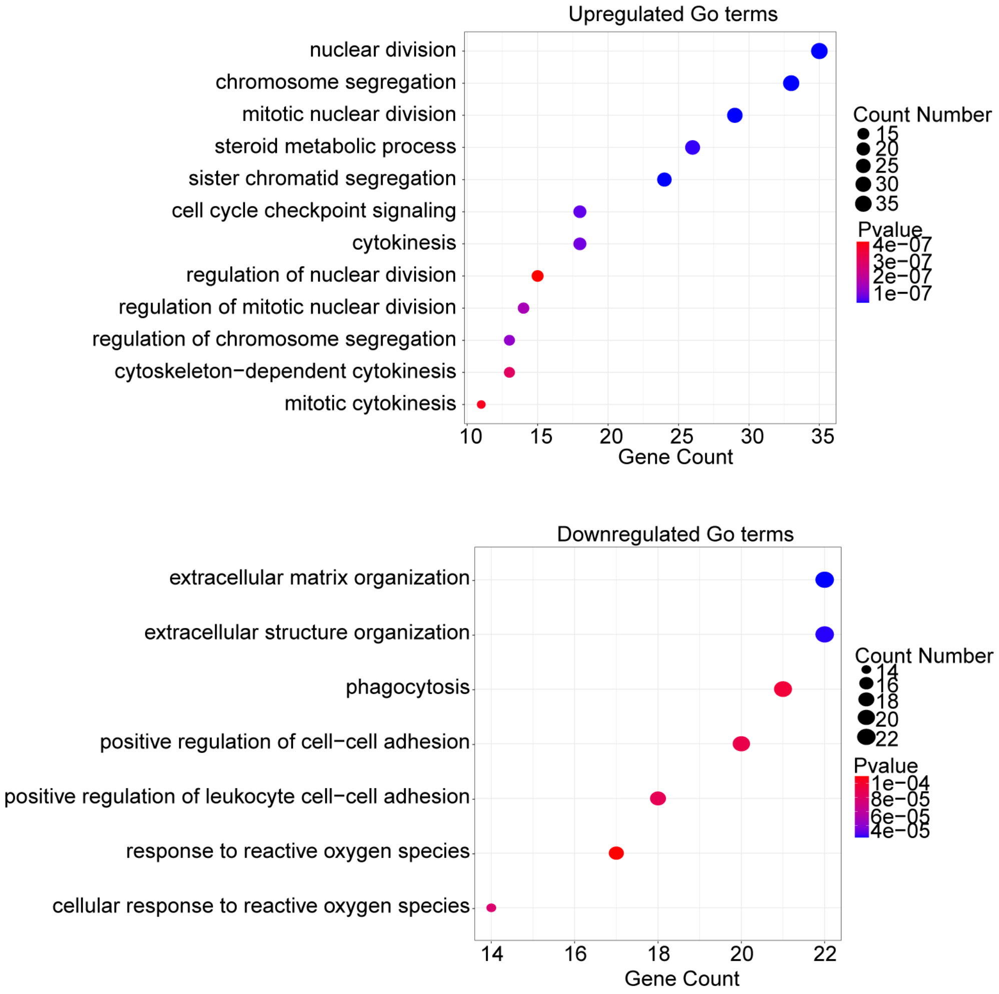

Figure S14. GO analysis of CHH treated with DMSO or 3C after injury , related to Figure S13

Supplemental figures legend

Figure S1. ScRNA-Seq analysis of hepatocytes isolated from healthy and CCl4-injured mouse livers，related to Figure 1

(A) Schematic of scRNA-Seq analysis of hepatocytes isolated from healthy and CCl4-injured mouse livers.

(B) UMAP visualization of hepatocytes from healthy (blue) and CCl4-treated (red) liver.

(C) The percentage of hepatocyte-enriched TFs (*Hnf1a*, *Hnf1b*, *Hnf4a*, *Foxa3*, *Foxa2*, *Foxa1*) and mesenchymal markers (*Col1a1*, *Col1a2*, *Acta2*, *Desmin*, *Pdgfra*, and *Pdgfrb*) expressing hepatocytes in total hepatocytes from healthy (blue) and CCl4-treated (red) liver based on scRNA-Seq.

(D) UMAP visualization of hepatocytes colored by the expression of hepatic genes *Alb*, *Arg1*, and *Tat,* and mesenchymal markers *Col1a1*, *Col1a2*, *Acta2*, *Desmin*, *Pdgfra*, and *Pdgfrb*.

(E) The percentage of inflammatory chemokine markers such as *Igfbp3*, *Cx3cl1*, *Ecm1*, *Lgals3*, apoptosis inhibiting factors (*Xiap*, *Birc2*, *Birc3*), expressing hepatocytes in total hepatocytes from healthy (blue) and CCl4-treated (red) liver based on scRNA-Seq.

(F) UMAP visualization of hepatocytes colored by the expression of senescence-associated secretory phenotype gene *Igfbp3*.

Figure S2. Characterization of dHeps from CCl4-induced mouse model by confocal analysis, related to Figure 1

(A) Schematic of the chronic liver fibrosis model (10% CCl4 injection twice weekly for 12 weeks) in the AAV-TBG-Cre system. CCl4 administration was initiated at 2 weeks after AAV injection and maintained for 12 weeks to induce chronic liver fibrosis.

(B) Z-stack confocal images of Desmin and Collagen in AAV-TBG-Cre: Rosa26-LSL-tdTomato lineage-tracing mice with and without CCl4 injury. Scale bar, 100 µm.

Figure S3. Characteristic comparison of isolated dHeps and healthy hepatocytes upon culture, related to Figure 1

(A) Relative mRNA expression of core hepatic TFs *Hnf4a* and *Hnf1a* for blank control (DMEM medium containing 10% FBS), base medium containing FSK or DMSO, and mouse adult liver.

(B) Hepatocytes isolated from 12 weeks CCl4-injured AAV-TBG-Cre: Rosa-LSL-tdTomato mice. Scale bar, 100 µm.

(C) Images showing morphology of hepatocytes isolated from CCl4-induced liver injury mice (CCl4) or healthy mouse liver (NC) labeled by tdTomato at day 0 and day16 of culture in hepatocyte culture medium *in vitro*.

(D, E) Morphology and percentage of Alb+ and Foxa2+ epithelial cells from livers of CCl4-injured (CCl4) and healthy (NC) mice at day 16 of growth in hepatocyte culture medium. Scale bar, 100 µm. Significance was assessed using unpaired Student's t- test.

(F) The mRNA expression of hepatic markers, mesenchymal identity, inflammatory-, apoptosis-, and senescence-related genes were detected by qPCR at 16 days of culture *in vitro*. Relative gene expression was normalized to the CCl4 group. n=2-3.

Figure S4. Gene expression analysis of dHeps isolated CCl4-induced mouse model treated with DMSO (D) or 5C for 16 days, related to Figure 1

(A) Hierarchical clustering heatmap based on the most variable genes among dHeps isolated CCl4-induced mouse model treated with DMSO (D) or 5C for 16 days. Log2 fold-change>1, p-value <0.05

(B) Gene Ontology (GO) analysis of upregulated and downregulated genes in dHeps isolated from a CCl4-induced mouse model treated with control (DMSO) or 5C for 16 days. Log2 fold-change>1, p-adjust <0.05. GO analysis indicated genes downregulated in the 5C group compared to the DMSO control cells were enriched for terms "phagocytosis", "inflammation", and "cytokine production", while upregulated genes were enriched for the terms "epithelial cell proliferation" and "metabolic processes".

(C) KEGG analysis of upregulated and downregulated genes in dHeps isolated from a CCl4-induced mouse model treated with DMSO (D) or 5C for 16 days. Log2 fold-change>1, p-adjust <0.05. KEGG pathway analysis showed that the upregulated differentially expressed genes (DEG) were enriched in "Bile secretion" and "Drug metabolism", while the downregulated DEGs were enriched in "NF-kappa B signaling pathway", and "Chemokine signaling pathway".

Figure S5. 5C induced the revitalization of hepatocytes injured by transforming growth factor β (TGF-β) treatment, related to Figure 2

(A, B) Representative images of morphology (A) and quantitative analysis of the number (B) of Hnf4a$^+$/Alb$^+$/tdTomato$^+$ hepatocytes cultured with 5C or DMSO on day 6 after treatment with or without TGF-β1 treatment. Results are means ± SD for three biological replicates. One-way ANOVA was performed to determine statistical significance. Scale bar, 100 μm. Positive controls healthy cells without TGF-β1 treatment (Null).

Figure S6. Characterization of Col1a2-CreER: Rosa-tdTomato labeling in mesenchymal cells after 5C treatment in healthy mice, related to Figure 4

(A) Experimental scheme for evaluating "leakiness" of the Col1a2 promoter activity in 5C-treated mice without liver injury.

(B) Immunofluorescence staining with antibodies (green) for Desmin, CYP3A4, and Hnf4a in cryosections mice expressing the Col1a2-CreER: Rosa26-LSL-tdTomato lineage-tracing system treated with 5C or solvent control. Nuclei are stained with DAPI (blue). Scale bar, 100 μm. n=3.

Figure S7. Restoration of regenerative potential in Col1a2-traced dHeps of mice with DDC-induced liver injury, related to Figure 4

(A) Schematic for generating a DDC-induced model of liver fibrosis to assess *in vivo* hepatocyte expansion of dHeps in Col1a2-CreER: Rosa26-LSL-tdTomato mice treated with 5C.
(B) Immunofluorescence staining with antibodies targeting hepatocyte markers CYP3A4 and Hnf4a in cryosections from 5C-treated and control mice expressing the Col1a2-CreER: Rosa26-LSL-tdTomato lineage-tracing system after DDC-induced liver injury. Scale bar, 100 μm.
(C) Numbers of tdTomato-expressing epithelial cell colonies per slide that stained positive for CYP3A4. Samples were obtained from the right liver lobe; n=3 mice per group. Significance was assessed using unpaired Student's t-test.

Figure S8. Absence of hepatocyte-like cells in organs other than the liver, related to Figure 4

(A) 1 month after 5C treatment, brain, heart, lung, muscle, spleen, and kidney were collected from Col1a2-CreER; Rosa26-tdTomato mice. Immunofluorescence staining showed the absence of CYP3A4 and Hnf4a positive hepatocyte-like cells in these organs. Scale bar, 100 μm.

Figure S9. Effect of 5C treatment on liver fibrosis in mice with DDC-induced liver injury mouse model, related to Figure 5

(A) Schematic for generating DDC-induced liver fibrosis model mice expressing Col1a2-CreER: Rosa26-LSL-tdTomato to assess the effect of 5C treatment or solvent control.

(B, C) H&E and Sirius red staining (B), which quantitative image analysis (C) to detect fibrosis in 5C-treated or control mice with DDC-induced liver fibrosis. Scale bar, 300 μm. Significance was assessed using an unpaired Student's t-test.

(D) Measurement of serum transaminases levels to assess *in vivo* liver function in 5C-treated liver injury mice (n = 3) relative to controls (n=3). Significance was assessed using unpaired Student's t-test.

Figure S10. The effect of 5C treatment in healthy mice, related to Figure 5

(A) The body weight of 5C-treated mice (n = 5) compared to controls (n = 5) for 4 weeks.
(B) H&E staining showed histological change of different organs in 5C-treated mice (n = 5) than in controls (n = 5). H&E, Scale bar, 200 μm.

Figure S11. GSK429286A, CID755673, and ETC-1002 (3C) were major contributors to hepatocyte revitalization, related to Figure 6

(A) Representative images of dHeps cultured in 5C with or without each component. GSK429286A, CID75567, ETC-1002, Salidroside, and Forskolin are represented in the diagram as G, C, E, S, and F, respectively. Scale bar, 100 μm.

(B) The effect of each small-molecule of 5C on the gene expression by single small-molecule omission assay. The mRNA expression of hepatic genes and fibrosis-associated factor was detected by qPCR for 16 days. Relative gene expression was normalized to 5C. n=2.

(C) Hierarchical clustering heatmap based on the most variable genes among dHeps isolated CCl4-induced mouse model treated with control (DMSO), 5C or 3C for 16 days. Log2 fold-change>1, p-value <0.05.

(D) Gene Ontology (GO) analysis of upregulated and downregulated genes in dHeps isolated from a CCl4-induced mouse model treated with control (DMSO) or 3C for 16 days. Log2 fold-change>1, p-adjust <0.05.

Figure S12. Chromatin accessibility analyses of chemical compounds to revitalize dHeps with identity loss, related to Figure 7

(A) Integrative Genomics Viewer (IGV) displaying ATAC-seq signals near representative hepatocyte-enriched TFs.

(B) Motif analysis of the open chromatic loci in the 3C group compared to the DMSO control, and closed chromatic loci in the hepatocytes isolated from CCl4 mouse model (CCl4) compared to the healthy liver (NC).

Figure S13. Chemical induction of hepatocyte revitalization in injured human hepatocytes

(A) After 5 days of treatment of 50ng/ml TGF-β1, cryopreserved human hepatocytes (CHH) were replaced with a base medium supplemented with 3C for 9 days. Hepatocytes treated with vehicle (DMSO) served as a control.

(B) Morphological changes and hepatic expression of ALB (Green) and CYP3A4 (Red) as determined by immunofluorescent staining in different treatments. Nuclei are stained with DAPI (blue). Scale bar, 100 μm.

(C) The number of ALB-positive hepatocyte-like cells per field for 3C and DMSO incubated human hepatocytes. Results are means ± SD for four biological replicates. Significance was assessed using one-way ANOVA.

(D) Hierarchical clustering heatmap based on the most variable genes among human hepatocytes treated with DMSO or 3C (EGC) for 3 days after 5 days of treatment of 50ng/mL TGF-β1. Day 0 represented in the diagram as the time point of TGFβ1 for 5 days. Log2 fold-change>0.5, p-value <0.05.

(E, F) Heatmaps showing the upregulation of genes associated with bile secretion, cell cycle, cell population proliferation, metabolic pathways, and the downregulation of genes associated with apoptosis, cellular senescence, collagen-containing extracellular matrix, necroptosis, and the TNF signaling pathway after treatment with 3C (EGC) compared with DMSO control. Day 0 represented in the diagram as the time point of TGF-β1 for 5 days. The color bar indicates gene expression in Log2 scale.

Figure S14. GO analysis of CHH treated with DMSO or 3C after injury, related to Figure S13

(A) GO analysis of upregulated and downregulated genes in CHH treated with DMSO or 3C for 3 days after 5 days of treatment of 50ng/mL TGF-β1. Log2 fold-change>1, p-value <0.05.

Supplemental table legend

Table S1. primers of qPCR, related to STAR Methods.

Table S1 primers of qPCR, related to STAR Methods

Gene	Forward	Reverse
(M) Gapdh	CCAATGTGTCCGTCGTGGAT	TGCCTGCTTCACCACCTTCT
(M) Foxa2	GGCCAGCGAGTTAAAGTATGC	TCATTCCAGCGCCCACATAG
(M) Hnf1a	AAAGCCGTGGTGGAGTCAC	ACAGGTGGGACTGGTTGAGA
(M) Alb	GCCACCATTTGAAAGGCCAG	TCACACCATCAAGCTTCGGG
(M) Hnf6	GGTCTGGGCAGCATTCACAAC	CAGGGTGGTGGGCTTCAAAG
(M) Hnf4a	TGACCATGGGCAATGACACG	TGTGGTTCTTCCTCACGCTC
(M) P16	AACTCTTTCGGTCGTACCCC	GCGTGCTTGAGCTGAAGCTA
(M) Acta2	GTCCCAGACATCAGGGAGTAA	TCGGATACTTCAGCGTCAGGA
(M) Vimentin	CGTCCACACGCACCTACAG	GGGGGATGAGGAATAGAGGCT
(M) Col1a1	GCTCCTCTTAGGGGCCACT	CCACGTCTCACCATTGGGG
(M) Cyp3a13	GACGATTCTTGCTTACCAGAAGG	CCGGTTTGTGAAGGTAGAGTAAC
(M) Ttr	CTGCTGTAGACGTGGCTGTAA	CTTCCAGTACGATTTGGTGTCC
(M) Cyp2b10	AAAGTCCCGTGGCAACTTCC	TTGGCTCAACGACAGCAACT
(M) Cyp2e1	GTTGCCTTGCTTGTCTGGAT	AGGAATTGGGAAAGGTCCTG
(M) Desmin	GTGGATGCAGCCACTCTAGC	TTAGCCGCGATGGTCTCATAC
(M) Timp1	GCAACTCGGACCTGGTCATAA	CGGCCCGTGATGAGAAACT
(M) Cd93	CAGAATGCAGCCGACAGCTA	GGCTGTACTGATTCTCCGGG
(M) Serpine1	TCCACAAGTCTGATGGCAGC	TGGTAGGGCAGTTCCACAAC
(M) Mmp3	TGCTGGTATGGAGCTTCTGC	CTGTCATCTCCAACCCGAGG
